# Identification of putative QTLs for seedling stage phosphorus starvation response in finger millet (*Eleusine coracana* L. Gaertn.) by association mapping and cross species synteny analysis

**DOI:** 10.1371/journal.pone.0183261

**Published:** 2017-08-18

**Authors:** M. Ramakrishnan, S. Antony Ceasar, K. K. Vinod, V. Duraipandiyan, T. P. Ajeesh Krishna, Hari D. Upadhyaya, N. A. Al-Dhabi, S. Ignacimuthu

**Affiliations:** 1 Division of Plant Biotechnology, Entomology Research Institute, Loyola College, Chennai, India; 2 Centre for Plant Sciences and School of Molecular and Cellular Biology, Faculty of Biological Sciences, University of Leeds, Leeds, United Kingdom; 3 ICAR-Indian Agricultural Research Institute, Rice Breeding and Genetics Research Centre, Aduthurai, Tamil Nadu, India; 4 Department of Botany and Microbiology, Addiriyah Chair for Environmental Studies, College of Science, King Saud University, Riyadh, Kingdom of Saudi Arabia; 5 International Crops Research Institute for the Semi-Arid Tropics (ICRISAT), Patancheru, Telangana, India; 6 The International Scientific Partnership Program (ISPP), King Saud University, Vice-19 Rectorate for Graduate studies and Research, Riyadh, Kingdom of Saudi Arabia; Aberystwyth University, UNITED KINGDOM

## Abstract

A germplasm assembly of 128 finger millet genotypes from 18 countries was evaluated for seedling-stage phosphorus (P) responses by growing them in P sufficient (P_*suf*_) and P deficient (P_*def*_) treatments. Majority of the genotypes showed adaptive responses to low P condition. Based on phenotype behaviour using the best linear unbiased predictors for each trait, genotypes were classified into, P responsive, low P tolerant and P non-responsive types. Based on the overall phenotype performance under P_*def*_, 10 genotypes were identified as low P tolerants. The low P tolerant genotypes were characterised by increased shoot and root length and increased root hair induction with longer root hairs under P_*def*_, than under P_*suf*_. Association mapping of P response traits using mixed linear models revealed four quantitative trait loci (QTLs). Two QTLs (*qLRDW*.*1* and *qLRDW*.*2*) for low P response affecting root dry weight explained over 10% phenotypic variation. *In silico* synteny analysis across grass genomes for these QTLs identified putative candidate genes such as *Ser-Thr kinase* and transcription factors such as *WRKY* and *basic helix-loop-helix* (*bHLH*). The QTLs for response under P_*suf*_ were mapped for traits such as shoot dry weight (*qHSDW*.*1*) and root length (*qHRL*.*1*). Putative associations of these QTLs over the syntenous regions on the grass genomes revealed proximity to *cytochrome P450*, *phosphate transporter* and *pectin methylesterase inhibitor* (*PMEI*) genes. This is the first report of the extent of phenotypic variability for P response in finger millet genotypes during seedling-stage, along with the QTLs and putative candidate genes associated with P starvation tolerance.

## Introduction

The major plant nutrient, Phosphorus (P) has a plentiful distribution in the soil, but is the most limiting nutrient, because of predominant P fixation [1–3], reaching up to 80% as organic P [4]. P is a unique nutrient element which forms the building block of most of the life bio-molecules. P nutrition has recently received strong focus for two contrasting reasons [5]; P deficiency is constantly on the rise worldwide, and excess P application in some areas has become a major socioeconomic concern due to environmental pollution [5]. Additionally, source of P fertilizers, natural rock phosphate, is declining at an alarming rate due to continuous mining [6] and may get exhausted in near future [7]. This has resulted in intermittent escalation of P fertilizer cost, pushing the poor and marginal farmers in the developing countries to resort to skipping of P fertilization [8].

To sustain agriculture under P scarce systems, it is imperative that P application should be reduced in the future. This may help in reducing P fertilizer requirement of crops, while helping to prevent environmental degradation [9] due to excess applied P, as well as in reducing anti-nutritional factors such as Phytate accumulation in grains that reduces the bioavailability of mineral elements such as Ca^2+^, Mg^2+^, Zn^2+^, Fe^2+^, Cu^2+^ and Mn^2+^ [10]. This can be achieved by improving the genetic potential of crop varieties to grow under P minimal conditions [11, 12], as well as to reduce grain phytate content [13–15].

Small millets, the earliest domesticated crop species of the world are heterogeneous group of cereals, often grown under harsher environments and subsist millions of poor people. They are nutritionally rich, genetically diverse and are recognised as crops for new green revolution [16] in the wake of impending climate change [17]. The finger millet (*Eleusine coracana* L. Gaertn.), a food staple of millions [18], is spread in about 12% of the global small millet area, across arid to semi-arid tropics of Asia and Africa [19]. It is rich in calcium content in contrast to rice and wheat [20]. Because of its nutritional prominence, genetic improvement of finger millet is a major breeding objectives worldwide [21]. Currently, this crop is being improved for calcium accumulation [22, 23] and nitrogen use efficiency [24–27]. However, efforts to improve tolerance to P deficiency have received less attention [28].

Using the recent molecular marker technology, development of genetic maps [29, 30] and mapping quantitative trait loci (QTLs) for traits such as morphological, agronomic and blast tolerance [31–34] has been reported in finger millet. Although reports from other cereals are available for P starvation tolerance [35, 36], no information on this is so far available in finger millet. P starvation response is a complex trait, and therefore, improvement of crop yield under low soil available P has been challenging due to various factors such as forms of P, intrinsic soil factors and environmental conditions. Despite this lacunae, P-related QTLs were reported by linkage mapping in rice [6], but only one QTL, *Pup1* (*Phosphorus uptake 1*) [37] is used for improving P starvation tolerance [12]. *Pup1* is located on chromosome 12 in rice, which harbours the key candidate gene *OsPSTOL1* coding for a Ser/Thr kinase protein that plays a key physiological role associated with crown root primordia in young seedlings enhancing early root growth and development [38]. Although *Pup1* does impart P starvation tolerance, it does not hold any P homeostasis related genes, implying that P homeostatic pathways are not the regulators for P starvation tolerance. Perhaps the external signals that drive the root system development do play a significant role in boosting P uptake from P limited soils [11].

Association mapping (AM) is a recent technique, to identify genomic regions in crops where linkage based mapping is still a challenge [39]. AM for different agro-morphological traits, protein and tryptophan contents and blast tolerance has been reported in finger millet [31–34]. AM uses linkage disequilibrium (LD) at the adjacent loci to locate the genomic regions associated with a trait, originated from evolutionary recombinations. By using genetic diversity and population structure along with their genetic relations, false discovery of marker trait association is controlled [40, 41]. Among other small millets, recently, QTLs for yield and other agronomic traits has been reported from foxtail millet [41] and for drought tolerance in pearl millet [42].

For improving P deficiency tolerance, identification of response traits that drive yield under P starved situations is crucial such as those related to the root system [43–45]. For instance, manipulation of root system architecture improves P foraging [36, 46] as observed in *Arabidopsis thaliana*, wherein increase in root hair density was reported under P starvation [47]. It is well established that a vigorous root system with enhanced nutrient uptake capabilities can lead to yield increase under optimized fertilizer management [6]. The major root architecture related traits for which QTLs have been reported are in crops such as rice [8, 48], corn [49, 50], soybean [51], wheat [52, 53], and common bean [54]. Nevertheless, target traits for low P tolerance can be different for different crops [55], as seen in the case of onion, wherein root system architecture seldom gets altered on exposure to P deficiency [56]. Since early crop establishment is crucial in crop success under nutrient limited conditions, the present study was aimed at mapping P deficiency responses in the finger millet genotypes at seedling stage. The germplasm assembly has diverse origin and had a distinct population structure [57]. The genotype responses were tested under two contrasting P levels and the microsatellite based genetic fingerprints were associated to identify marker-phenotype association.

## Materials and methods

### Plant material

One hundred and twenty-eight finger millet genotypes from major centres of diversity (India, Uganda, Zimbabwe, Germany, Malawi, USA, Nepal, Kenya, Burundi, Nigeria, Malaya, Maldives, Tanzania, Somalia, Tanganyika, Ethiopia, Senegal and Sri Lanka) were collected from the International Crops Research Institute for the Semi-Arid Tropics (ICRISAT), University of Agricultural Sciences (UAS) Bangalore, and Tamil Nadu Agricultural University (TNAU), Coimbatore. The details of 128 genotypes of finger millet and their origins can be found in Ramakrishnan et al. [58].

### Phenotyping under contrasting P levels

The genotypes were grown in horticultural grade perlite filled plastic pots (23 cm diameter at top and 14 cm diameter at bottom with 20 cm depth) for 30 days. Two levels of P were maintained, the P_*def*_ containing 0.3 ppm of P (10 μM of KH_2_PO_4_) and the P_*suf*_ having 9.3 ppm of P (300 μM of KH_2_PO_4_) in nutrient solution. The remaining composition of the nutrient solution was kept constant, and contained 0.1 mM KCl, 0.1 mM K_2_SO_4_, 2.0 mM Ca(NO_3_)_2_, 0.5 mM MgSO_4_, 0.5 μM MnCl_2_, 0.5 μM ZnCl_2_, 0.2 μM CuCl_2_, 10 μM H_3_BO_3_, 0.1 μM Na_2_MoO_4_ and 0.1 mM Fe-EDTA prepared and diluted using demineralised water [59]. The pH of the solutions was adjusted to 6.0 using 0.1 M H_2_SO_4_ or 0.1 M NaOH. The perlite filled pots were irrigated with 500 ml of nutrient solution once in four days. The freshly harvested seeds of the genotypes were surface sterilised by immersing in 0.5% sodium hypochlorite (NaOCl) solution for 3 minutes. The seeds were then washed thoroughly using demineralised water several times and sown directly in the pots. After germination, the seedlings were thinned to maintain a population of 15 seedlings per pot. The experiment was conducted in the greenhouse at Entomology Research Institute, Loyola College, Chennai, during March-June 2015. The green house was maintained with 27 ± 2°C and 85% relative humidity under well-lit and aerated conditions. Three replications were used for each genotype for two P concentrations.

Three 15-day old uniform sized seedlings from each pot were carefully extracted with intact roots, washed with demineralised water and blotted dry using lint free filter paper. After separating shoot and root portions using a fine scissor, the shoots and roots were then placed separately in paper sleeves and dried for 72 hours in a hot air oven at 65°C. At the end, shoot and root dry weights (SDW and RDW respectively) were determined. After 30 days of growth, three uniform looking seedlings per pot were carefully extracted intact, and the roots were cleaned free of perlite granules and washed with demineralised water. For the measurement of root length (RL), the roots were blotted dry using lint free filter paper and carefully stretched over a stainless steel ruler using forceps and the length was measured. Similarly, shoot length (SL) was also measured immediately after extraction from the pots as the distance between collar and shoot tip. Root portions of the seedlings were separated and preserved in de-ionized water immediately after the measurement of SL and RL. Root hair measurements were based on the method earlier described [60] with some minor modifications. To improve the precision, pot culture was repeated thrice. Since the data were consistent across the repeats, average data was used for further analysis.

About 5 cm from the primary root cap was chosen for root hair analysis in all genotypes. The root portions were placed on a stage micrometre with a scale (10 μm) and observed in a Stereo Microscope (Leica Stereo Microscope; Wetzlar, Germany) with 10x magnification. The images were captured with the help of a digital camera (Sony CyberShot DSC-WX200). The length of root hairs and density were counted using ImageJ Scientific Software [61].

### Statistical analyses

Descriptive statistics were computed for phenotypic traits under both P_*def*_ and P_*suf*_ treatments. Analysis of variance (ANOVA) was performed using a mixed model, in which genotypes were taken as fixed and the P levels as random. Based on the traits that had significant genotype x P level interaction, using the best linear unbiased predictors (BLUP) for genotype-by-P level means, genotype behaviours under P levels were empirically grouped as P responsive, low P tolerant and P non-responsive for individual traits. The frequency of genotypes that deviated from the upper tail value critical difference was taken as P responsive, and those deviated from the lower tail value were taken as low P tolerant, based on the relative deviation computed in percentage based on the performance under P_*def*_. The intermediate behaviours shown by the genotypes falling within the upper and lower tail limits were taken as P non-responsive. The shoot and root traits that were taken on 15 days after germination were dropped from further analysis to ascertain low P tolerants. Since the major focus of the study was on identification of low P tolerance of genotypes after 30 days of germination, a graphical comparison of list of genotypes showing low P tolerant behaviour for the traits SL, RL, RHD and RHL, was done using a Venn diagram drawn in VennPainter 1.2.0 [62]. Correlation coefficients were also determined among different traits using the BLUPs.

### Genotyping and population structure

The genomic DNA was extracted from young leaves of finger millet genotypes using Doyle and Doyle [63] method slightly modified by Ramakrishnan et al. [58]. Genotyping was performed using 72 polymorphic SSR markers designed from the finger millet accession PI 321125 through the random genomic libraries generated from the *Hind*III, *Sal*I and *Pst*I restriction digests through probe hybridisation [29].

The genotyping data from the test accessions were analysed to determine the population structure using a model-based Bayesian statistics implemented to subdivide genotypes into genetic sub-populations (SPs) using the software STRUCTURE v.2.3.4 [64, 65]. No prior information was used to determine SPs and it had been expected that the population is structured, because of the diverse origin of the members of the panel. However, an admixture model was assumed with correlated allele frequencies with implication of migration from a common centre of origin. The proposed model was run by considering a population substructure (*K*) ranging from 1 to 10 with three independent runs per *K*. The model was run with 100000 Markov Chain Monte Carlo (MCMC) simulations [66] preceded by a burn-in length of 100000 to bring in the unbiasedness of the starting point which is a representative of the equilibrium distribution [67]. The optimum *K* was determined by an ad hoc statistic Δ*K* which is a ratio (modal value) of the absolute value of the rate of change of the mean log likelihood, LnP(*K*) between sequential *K* values to its standard deviation [68]. The *K* with tallest Δ*K* is selected as the optimum *K*. Parsing of the results of Structure was done through the online version of the program Structure Harvester web v0.6.94 [69].

### Association mapping

The sub-population membership coefficients (inferred ancestry) of the genotypes for the three significant sub-populations (*k* = 3) were used as the Q-matrix for AM. The finger millet genotypes’ genetic relatedness was calculated as kinship by weighing identical by state (IBS) of the common alleles among the accessions [70] through the software TASSEL v5.2 [71]. The genotypes were scored as 2, 1, or 0 equal to the count of one of the alleles at that locus. The missing genotypes were assigned using average genotype score. The score data estimated the relationship matrix.

AM of genotype and phenotype data was performed to identify robust marker-trait association using the software TASSEL v5.2, following mixed linear model (MLM) approach [71]. Since MLM method showed better false association control than general linear model (GLM) method [72], the AM was restricted to MLM alone. The significant threshold for valid QTLs-trait association was determined by applying a Bonferroni correction by dividing the alpha of 0.05 by the number of markers. The p-values lower than the computed threshold was used for identifying valid QTLs [73]. In addition, a multi-locus mixed model (MLMM) association was carried out using a forward step wise approach [74] to obtain consensus associations between different methods. The analysis was performed using SVS v8.7 (GoldenHelix® Software).

### Cross genome synteny search

Since the whole genome sequence of finger millet is not yet available, we have used cross genome synteny search for orthologous regions, an *in silico* comparative genomics approach, to explore the identified QTLs for candidate gene references. Nucleotide basic local alignment search tool (nBLAST) was carried across ten cereal genomes included in Phytozome v. 11.0 [75] to carry out the sequence alignment search using the original finger millet genome sequences from which the microsatellite markers have been sourced [29]. The original random genomic library sequences corresponding to each QTL linked marker obtained from Dr Ketrien Devos, University of Georgia, were used as the search key. The length of each library sequence was 1164 bp (UGEP19), 1260 bp (UGEP13), 1203 bp (UGEP68), and 1544 bp (UGEP90). BLAST engines are designed to search for a minimum of 22 nucleotide sequences or 6 amino acid sequences. Significant hits were taken based on maximum threshold; E-value of 0.01 was empirically fixed during the search to pick potentially coding elements [76] for the full length of target sequence. The sequence alignment hits obtained on the cereal genomes were located on the chromosomes of corresponding species, and analysed for the presence of annotated candidate gene sequences near the query sequence. The functions of such closely associated putative genes were further analysed for their significance to P starvation response. To identify the biochemical pathway in which the candidate genes are involved, an extensive search was conducted in the Kyoto Encyclopaedia of Genes and Genomes (KEGG, http://www.kegg.jp) database [77]. The positive hits of the searched genes were related to P starvation response in the germplasm.

## Results

### Agro-morphological response under different P levels

All the genotypes germinated within 4–5 days of sowing in the perlite filled plastic pots both under P_*def*_ and P_*suf*_ treatments. ANOVA showed significant genotypic variation within each level by single environment analysis, but combined (multi-environment) analysis revealed significant genotype x P level interaction for all the traits, and non-significant interaction for genotype and P level effects for most of the traits excluding SDW, RL, RHD and RHL (**Table 1**). At 15 days after germination, P_*def*_ had significantly lower values for SDW and RDW (**Table 2**). However, after 30 days of germination, under P_*def*_, average SL and RHD increased while RL decreased than under P_*suf*_. Similarly, RHL was also higher under P_*def*_ as compared to P_*suf*_. On single environment analysis, coefficient of variation under the P levels was high for all the traits. The mean performance of all the genotypes under two P levels is provided in **S1 Table**.

**Table 1:** Analysis of variance for testing the significance for genotype, P level and interaction effects using linear mixed model.

Traits	Phenotypic variance (fixed effect)			Chi Square values (random effect)	
	P_*suf*_	P_*def*_	Pooled	P level	Genotype x P level
SDW	15.06**	5.90**	4.09*	8.60**	77.26**
RDW	1.71**	0.98**	0.75*	2.90^ns^	32.50**
SL	24.49**	24.81**	3.88^ns^	2.65^ns^	234.06**
RL	20.51**	11.74**	4.08 ^ns^	11.22**	133.53**
RHD	151.92**	122.98**	23.67*	15.16**	249.79**
RHL	17.84**	10.35**	0.90^ns^	23.34**	519.71**

^ns^, non-significant

*; significant at *p*<0.05

**, significant at *p*<0.01

SDW, shoot dry weight in g; RDW, root dry weight in g; SL, shoot length in cm; RL, root length in cm; RHD, root hair density per 10 μm primary root length; RHL, root hair length in μm

**Table 2:** Candidate traits variation under P_*suf*_ and P_*def*_ treatments among 128 genotypes of finger millet.

Traits	P_*suf*_				P_*def*_			
	Mean	Range	CV %	SE	Mean	Range	CV %	SE
SDW (mg)	4.5	1.2–15.3	60.1	1.53	3.5	1.0–8.3	51.6	1.16
RDW (mg)	1.9	0.7–5.0	52.7	0.68	1.7	0.7–4.2	43.9	0.49
SL (cm)	6.9	2.5–21.4	47.7	1.56	8.4	4.5–22.2	39.5	1.58
RL (cm)	9.9	4.1–21.5	31.4	1.70	8.1	3.5–17.9	29.4	1.35
RHD	23.7	7.7–39.7	33.2	3.38	31.0	15.3–48.7	23.0	3.18
RHL (μm)	7.4	2.3–14.7	34.6	0.75	9.6	4.0–14.7	21.0	0.81

CV, coefficient of variation; SE, standard error; SDW, shoot dry weights; RDW, root dry weights; SL, shoot length; RL, root length; RHD, root hair density per 10 μm primary root length; RHL, root hair length

The SDW recorded under P_*suf*_ showed the genotype IE2606 (15.3 mg) having highest dry weight. This was followed by the genotype MR2 (14.4 mg). The mean dry weight among all the genotypes was 4.51 (**Table 2**). On the other hand, under P_*def*_, the mean SDW recorded was 3.53 mg among all the genotypes, with IE3104 recording the highest dry weight of 8.3 mg. The RDW also showed similar trend as SDW, having a range of 0.7–5.0 mg with a mean value of 1.9 mg among all the genotypes under P_*suf*_. Under P_*def*_, RDW ranged between 0.7 and 4.2 mg with a mean value of 1.7 mg. Four genotypes MR2 (5.0 mg), IE7018 (4.4 mg), IE2606 (4.2 mg) and IE4734 (4.1 mg) recorded high RDW under P_*suf*_ while two genotypes IE5106 (4.2 mg) and IE3104 (4.0 mg) showed higher RDW under P_*def*_ (**S1 Table**). SDW showed significant positive correlations with RDW, SL and RL under both the P levels, while RDW was found correlated to SL alone.

The RL decreased significantly under P_*def*_ with a mean length of 8.1 cm, as against 9.9 cm under P_*suf*_. SL was more under P_*def*_ recording a mean of 8.4 cm, while the average SL under P_*suf*_ level was 6.9 cm. The RL under P_*suf*_ ranged from 4.1 cm (IE3945) to 21.5 cm (MR2) with a mean value of 9.9 cm, whereas under P_*def*_, RL values were in the range of 3.5 cm (KRI00701) to 17.9 cm (RAU8) with a mean of 8.1 cm per plant (**Table 2**). Other than RAU8, TCUM1 (17.6 cm) produced longer roots under P_*def*_. P_*suf*_ also supressed RL in genotype IE3945 (4.1 cm), followed by IE3618 (4.8 cm), SVK-1 (5.4 cm) and each GPU-28 and VL149 (6.0 cm). Under P_*suf*,_ only one genotype MR2 (21.5 cm) showed maximum RL, which also produced the longest shoot (21.4 cm) under P_*suf*_. Under P_*def*_, IE6350 produced shoot of 22.2 cm length (**S1 Table**).

**Fig 1:**
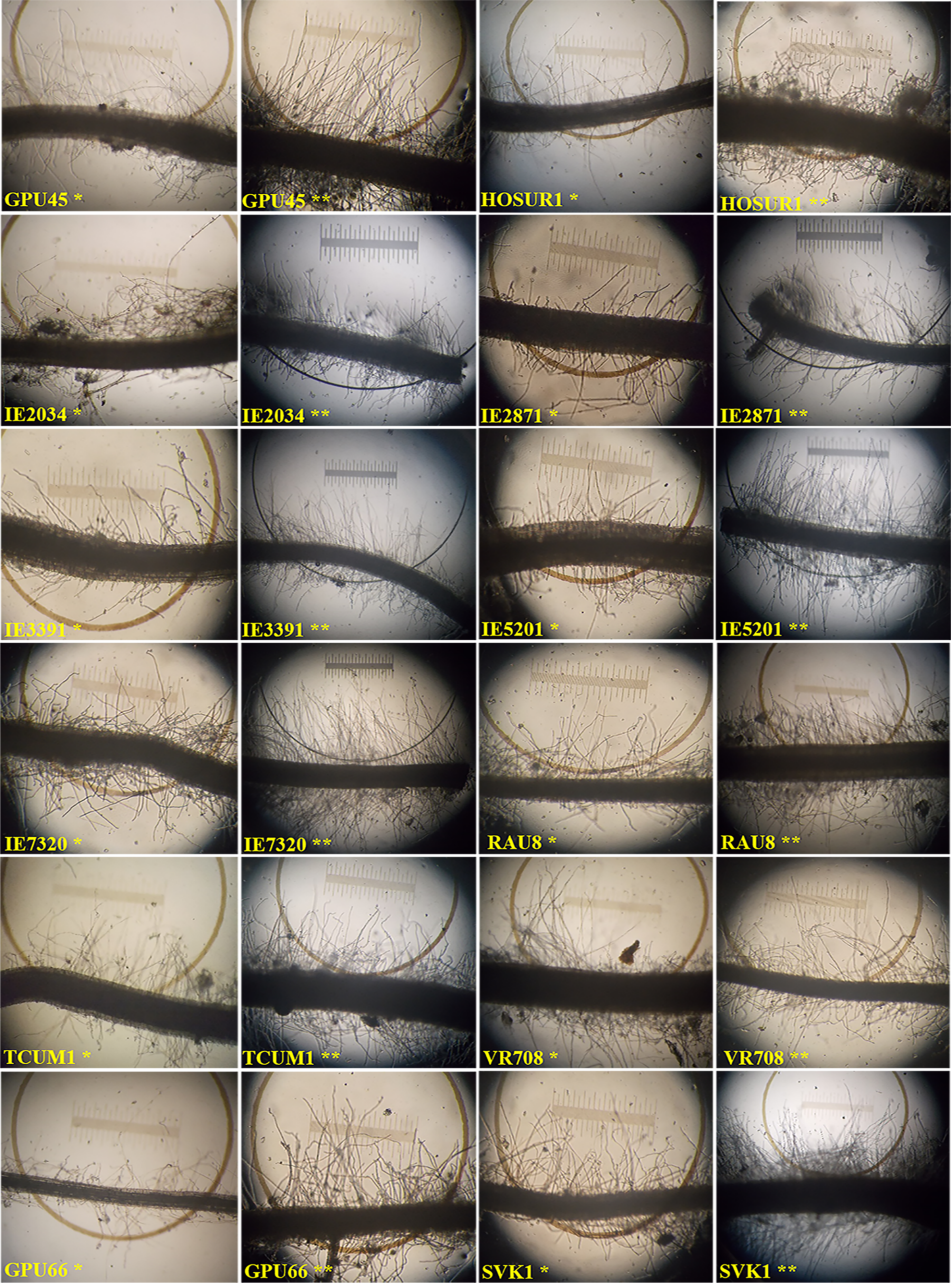
Root hair responses in the selected low P responding finger millet genotypes showing low and high number of root hairs under P_*suf*_ (*) and P_*def*_ (**) conditions respectively.

The genotypes under P_*def*_ produced more root hairs than under P_*suf*_, which was observed from high values for RHD. The RHD ranged from 7.7 to 39.7 with a mean value of 23.7 per 10 μm length of primary roots under P_*suf*_ as against the range of 15.3 to 48.7 with a mean value of 31.0 under P_*def*_ level (**Table 2**). Under P_*def*_ level, MR6 (48.7), IE4491 (46.3), IE5066 (45.7) and L5 (45.3) were the top genotypes that recorded highest RHD, while under P_*suf*_, the genotype IE4816 (39.7) followed by APSKK1 (38.7) produced better response for RHD. The genotypic response for RHL was similar to that of RHD, where longer root hairs were produced under P_*def*_ than under P_*suf*_ (**Fig 1**). RHL ranged from 4.0 to 14.7 μm under P_*def*_ with a mean value of 9.6 μm, wherein RHL ranged from 2.3 to 14.7 μm with an average value of 7.4 μm under P_*suf*_. The genotype IE2821 recorded an RHL of 14.7 μm under P_*suf*_, whereas IE7320 produced similar RHL under P_*def*_ (**S1 Table**). There was no correlation between RHD and RHL. For example, the genotype MR6 produced maximum RHD (48.6) but the highest RHL was obtained in genotype IE7320 (14.7 μm). Similar trend was obtained for other genotypes.

### Empirical classification of genotypes based on P response

Comparisons based on the relative deviation of the BLUPs of each genotype’s performance under P_*def*_ and P_*suf*_ treatments were done empirically to classify genotype behaviours as P responsive, P non-responsive and low P tolerant (**Table 3**). In grouping based on SDW, there were 89 P responsive genotypes and 18 low P tolerant followed by 21 P non-responsive ones. For RDW, 79 P responsive genotypes, 30 low P tolerants and 19 P non-responsive genotypes were identified.

**Table 3:** Genotype behavior classes based on the BLUPs for genotype x P level interaction response on different traits (The values in parenthesis show membership percentage in each class).

Behaviour class	SDW	RDW	SL	RL	RHD	RHL
P responsive	89.00 (69.53)	79.00 (61.72)	32.00 (25.00)	96.00 (75.00)	17.00 (13.28)	22.00 (17.19)
Low P tolerant	18.00 (14.06)	30.00 (23.44)	85.00 (66.41)	22.00 (17.19)	105.00 (82.03)	99.00 (77.34)
P non-responsive	21.00 (16.41)	19.00 (14.84)	11.00 (8.59)	10.00 (7.81)	6.00 (4.69)	7.00 (5.47)
CD (p<0.05)	7.50	4.34	6.22	5.28	3.80	5.19

SE, standard error; SDW, shoot dry weight; RDW, root dry weight; RL, root length; RHD, root hair density; RHL, root hair length; CD, critical difference

The remaining four traits RL, SL, RHD and RHL observed after 30 days of germination showed almost similar pattern in the genotype classification. As a general response behaviour in several genotypes, SL, RHD and RHL increased while RL decreased under P starvation. There were 32 P responsive genotypes that produced longer shoot under P_*suf*_, 85 low P tolerant ones which had longer shoots under P_*def*_ and 11 P non-responsive genotypes, which did not show significant variation in SL under both P levels (**Table 3**). For RL, there were 96 genotypes that produced shorter root length under P_*def*_ that were recognised as P responsive ones; and of the remaining 32 genotypes, 22 produced longer roots under P_*def*_ which were identified as low P tolerant and remaining 10 as P non-responsive genotypes. Similarly, 17 P responsive genotypes were identified that had higher RHD under P_*suf*_, whereas 105 genotypes had high RHD under P_*def*_, while 6 of them did not show significant variation for RHD under both the P levels. For RHL, 99 were identified as low P tolerant genotypes, 22 as P responsive and 7 as P non-responsive genotypes (**Table 3**).

**Fig 2:**
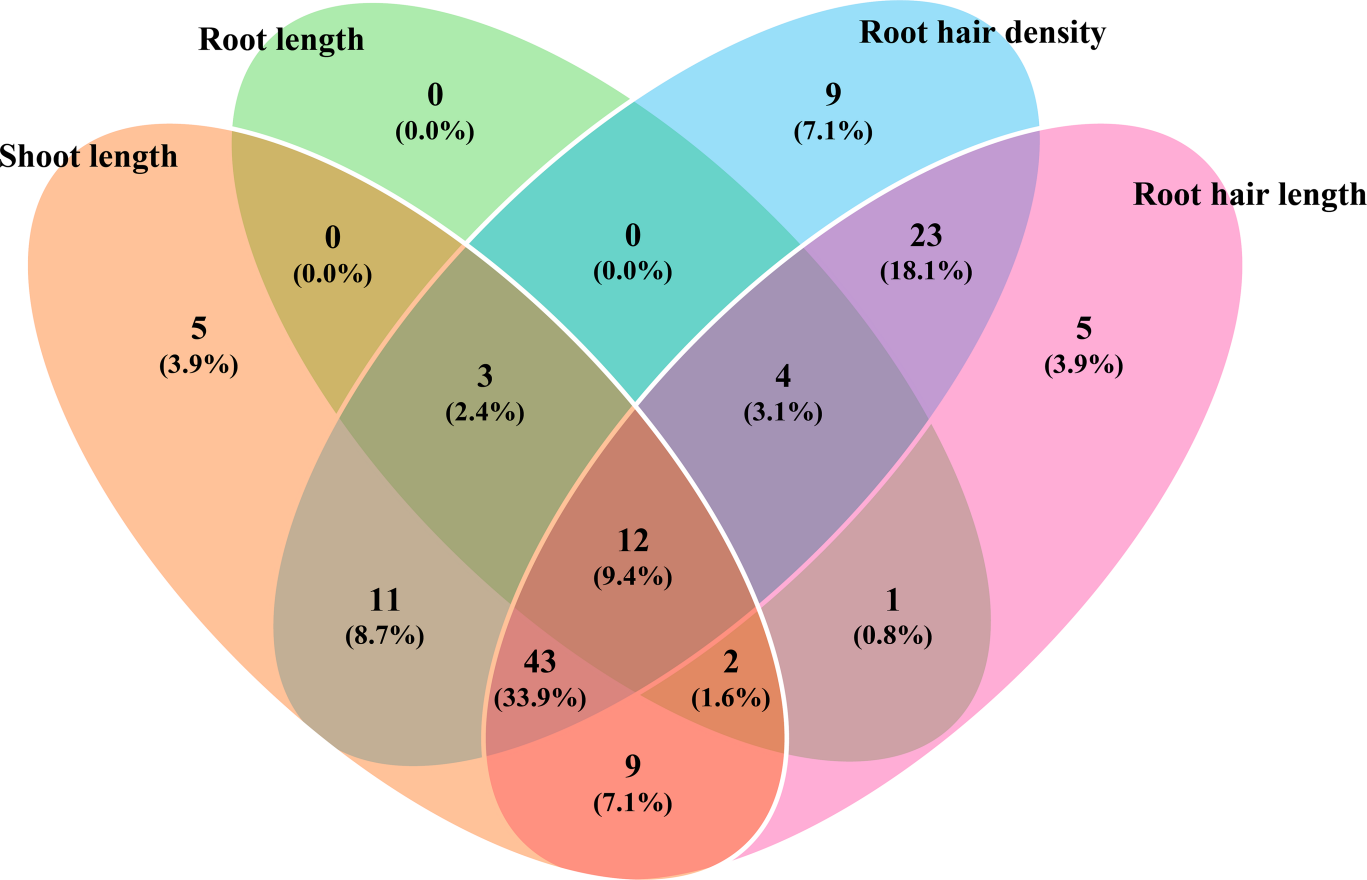
Venn diagram comparing the genotype list based on low P tolerance exhibited for traits SL, RL, RHD and RHL. The graphical comparison was analyzed based on genotypes’ performance for SL, RL, RHD and RHL under P_*def*_. The SDW and RDW were not used for graphical comparison. The graphical comparison identified 12 (9.40%) genotypes as low P tolerants, which produced higher values for traits SL, RL, RHD and RHL.

The graphical comparison of lists of genotypes that were low P tolerant for traits SL, RL, RHD and RHL identified 12 common genotypes constituting 9.40% of the total genotypes that were low P tolerant based on all the lists, those which produced higher values for all the traits under P_*def*_ (**Fig 2**). These 12 genotypes were identified as low P tolerant genotypes. The root hair images of all the low P tolerant genotypes are shown in **Fig 1**. The remaining genotypes shared between different list combinations (**Table 4)**. Among these, there were large groups of 43 genotypes that had high values for SL, RHD and RHL, among which 39 had low values for RL under P_*def*_ (**S2 Table**). The remaining four genotypes were either showing intermediate or non-responding behaviour for RL.

**Table 4:** Genotypes having low P tolerance responses with different combinations of traits for positive responses for all the traits.

Trait combinations	Members	Genotype
RHD, RHL, RL, SL	12	GPU45, IE5201, IE2871, IE7320, GPU66, HOSUR1, TCUM1, IE2034, SVK1, RAU8, VR708, IE3391
RHD, RL, SL	3	IE6326, IE3945, IE3475
RHL, RL, SL	2	IE6337, GPU28
RHD, RHL, SL	43	IE5106, IE2043, PR202, IE4057, IE2457, VL149, GPU46, IE6240, IE2589, VIJAYAWADA, ML365, IE6514, KRI00701, IE4797, IE4622, PAIYUR2, IE5367, IE2437, IE2957, MR6, IE4545, IE5306, IE5817, IE4671, IE501, KM252, IE6082, TRY1, L5, IE2042, IE5870, INDOF7, IE2572, IE4757, INDOF9, IE3470, IE6350, INDOF5, IE4491, IE4570, IE3045, IE3392, IE5066
RHD, RHL, RL	4	IE6221, IE4646, CO11, IE3618
RHD, SL	9	IE4816, IE2911, IE2872, IE2790, THRVP, IE4121, IE5091, IE7079, IE4709
RHL, SL	1	CO14
RHL, RL	23	HR911, IE4673, MR2, IE4795, IE6473, IE5537, IE2217, IE3973, IE7018, GPU26, IE2606, IE2619, IE6059, IE2430, IE4329, THRP1, KMR301, IE4497, IE518, TCHIN1, INDOF8, KRI1311, IE4028
RHD, RHL	5	IE2710, CO12, IE6537, IE1055, GPU48
SL	9	IE4734, CONO1, IE3614, IE6165, APSSK1, DPI00904, IE6421, HR374, CO9
RHD	5	IE2296, THRVPP, IE3721, IE2312, IE3317
RHL	12	GPU45, IE5201, IE2871, IE7320, GPU66, HOSUR1, TCUM1, IE2034, SVK1, RAU8, VR708, IE3391

SL, shoot length; RL, root length; RHD, root hair density per 10 μm primary root length; RHL, root hair length

### Inter-trait associations

Interrelations of same trait between two P levels showed significant association only for few traits such as SDW, RDW, RL and RHD (**Table 5**). No association for SL and RHL was found between P levels. Across P levels, SDW was found positively and significantly correlated with RDW and RL. Few other significant associations were observed only under P_*suf*_, leaving no other significant traits association under P_*def*_. Under P_*suf*_, SDW was found correlated to RDW. Similar were the associations of SDW with SL and RDW with SL and RL. SL was also found related to RL; RL showed a significant negative association with RHL under P_*suf*_.

**Table 5:** Pearson’s correlations among the predicted trait means under P_*suf*_ (lower diagonal) and P_*def*_ (upper diagonal) conditions. The diagonal values (bold) are the correlations between the P levels.

	Trait	P_*def*_					
		SDW	RDW	SL	RL	RHD	RHL
**P**_***suf***_	**SDW**	0.574*	0.619*	0.097	0.277*	0.124	0.060
	**RDW**	0.784*	0.688*	0.081	0.182	0.078	0.029
	**SL**	0.446*	0.421*	**0.191**	0.060	0.114	0.064
	**RL**	0.387*	0.368*	0.447*	0.340*	0.090	0.006
	**RHD**	0.089	0.141	0.059	-0.001	0.336*	0.198
	**RHL**	-0.035	0.034	-0.145	-0.230*	-0.036	**0.064**

SDW, shoot dry weight; RDW, root dry weight; RL, root length; RHD, root hair density; RHL, root hair length

* significant at *p*<0.01

### Population structure

**Fig 3:**
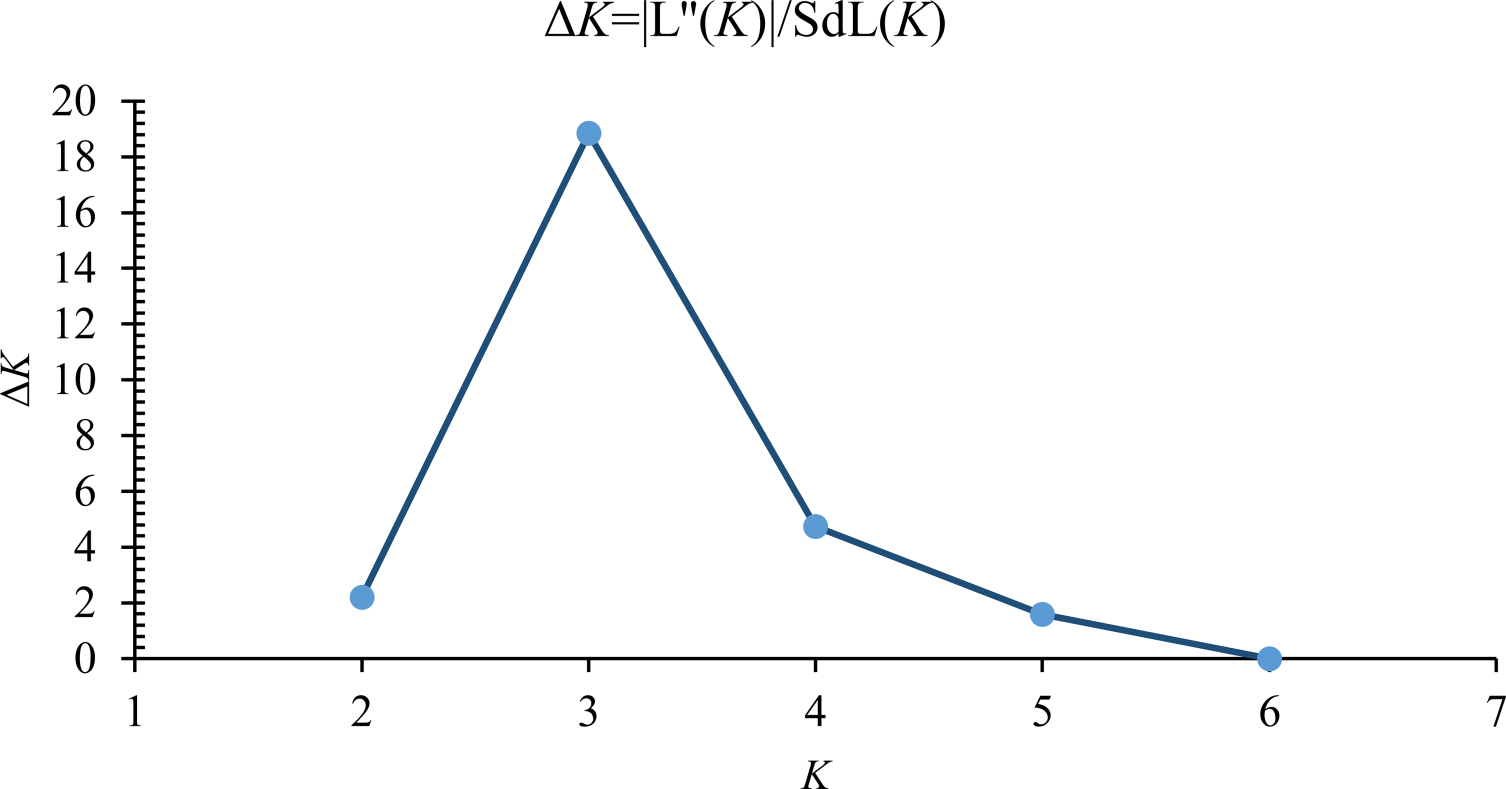
Identification of optimum population structure using Evanno’s method. The Δ*K* values showed the highest peak corresponding to *K* = 3.

**Fig 4:**
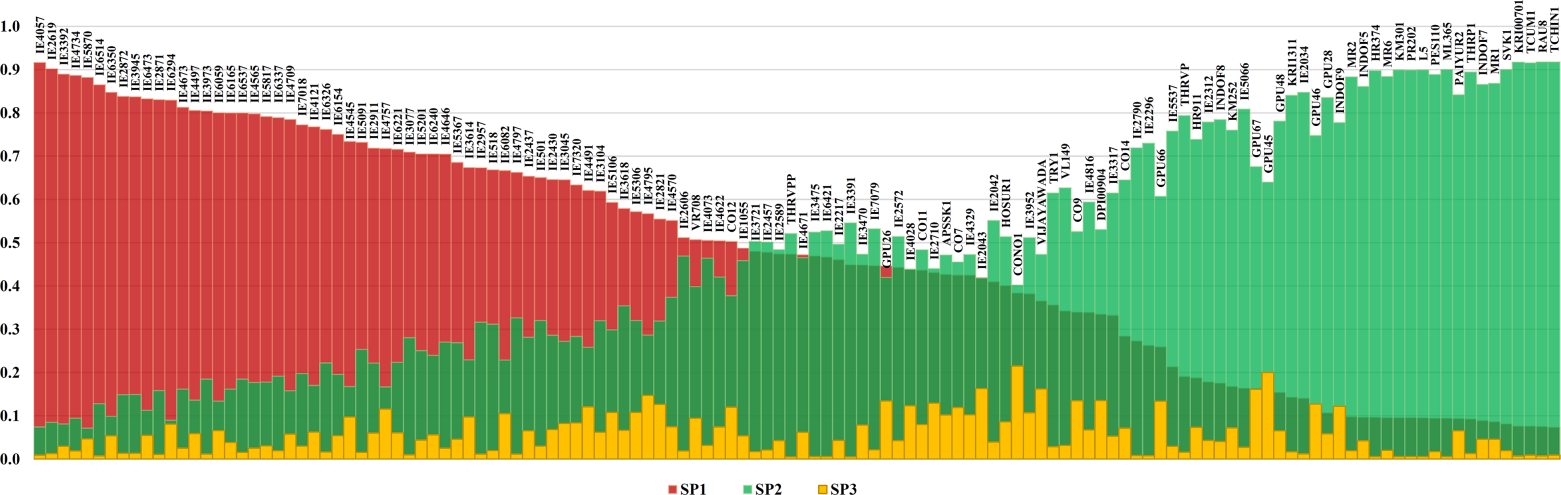
Inferred ancestry coefficients of the genotypes forming the sub-populations, SP1, SP2 and SP3.

The population structure analysis indicated that the maximum Δ*K* value determined was *K* = 3 (**Fig 3**) which showed that the 128 finger millet genotypes broadly grouped into three SPs (SP1, SP2 and SP3). SP1 was found to contain exotic germplasm prominently while SP2 was predominated with indigenous collections. The pattern of genetic differentiation between SPs revealed that SP3 was admixture of the first two SPs (**Fig 4**). The genetic relationship showed various confirmations for gene flow between SPs. The expected heterozygosity of the SP1 was maximum (0.48) followed by SP2 and SP3. The membership proportions of SP1 and SP2 were 48 and 47% respectively, while SP3 had 5% of the population. The allele frequency divergence between SP1 with SP2 and SP3 was 0.106 and 0.102 respectively, while SP2 and SP3 had divergence of 0.002.

### Identification of QTLs for P tolerance by association mapping

AM identified four QTLs (markers) associated with three candidate traits namely RDW under P_*def*_ and SDW and RL under P_*suf*_ (**Table 6**). No other trait was significantly associated with marker data. The QTL, *qLRDW*.*1* associated with RDW under P_*def*_ was relatively stronger than other QTLs, explaining 14.3% of the phenotype variation for this trait. This was associated with the marker UGEP19 with a very low probability of 4.69 x 10^−5^. The other QTL identified for RDW under P_*def*_, *qLRDW*.*2*, was linked to marker UGEP68 and reported a proportion of 10.56% of the phenotype variation. Two other QTLs, identified for the responses under P_*suf*_, were related to SDW and RL. The QTL, *qHSDW*.*1* was associated with UGEP13 at a probability of 8.23 x1 0^−5^ and accounted for 12.71% of the phenotype variation for this trait. Further, the QTL, *qHRL*.*1* linked to UGEP90 was associated to the RL, and explained 9.23% of the total phenotype variation (**Table 6**). Although, these four markers generated different alleles, the QTL effects were identified only for specific alleles in each case. For marker UGEP19, the allele of size 226 bp only was found associated with the response under low P condition. Similarly, the allele of size 234 bp for UGEP68 was found associated with the same trait. For other markers, the allele 208 bp of UGEP13 was associated with *qHSDW*.*1*, while *qHRL*.*1* was associated with 228 bp allele of UGEP90. Although MLMM procedure reduced the estimated probability for two QTLs, *qLRDW*.*2* and *qHRL*.*1* significantly, all four QTLs were identified in both the methods.

**Table 6:** SSR markers associated with candidate traits using MLM and MLMM based association mapping in 128 finger millet genotypes.

Trait	Marker	QTL	Allele Size (bp)	R^2^ (%)	p	V_G_
***Mixed linear model mapping (MLM)***						
LRDW	UGEP19	*qLRDW*.*1*	226	14.3	4.69E-05	0.33
LRDW	UGEP68	*qLRDW*.*2*	234	10.6	4.19E-04	4.81
HSDW	UGEP13	*qHSDW*.*1*	208	12.7	8.23E-05	0.33
HRL	UGEP90	*qHRL*.*1*	228	9.2	6.32E-04	6.43
***Multiple linear mixed model mapping (MLMM)***						
LRDW	UGEP19	*qLRDW*.*1*	226	9.5	8.92E-05	0.06
HSDW	UGEP13	*qHSDW*.*1*	208	12.7	1.53E-4	5.22E-5
LRDW	UGEP68	*qLRDW*.*2*	234	6.5	1.05E-3	0.61
HRL	UGEP90	*qHRL*.*1*	228	9.2	1.06E-3	2.94

HSDW, shoot dry weight under P_*suf*_ level; LRDW, root dry weight under P_*def*_ level; HRL, root length under P_*suf*_ level, F, variance ratio; R^2^, phenotypic variation explained; p, marker probability

### Exploring candidate genes by cross genome synteny

The original sequences of microsatellite regions associated with the QTLs were observed to be syntenous (orthologues) on genomes of ten species from grass family such as *O*. *sativa*, *Brachypodium distachyon* (Purple false brome), *B*. *stacei* (Purple false brome), *Panicum hallii* (Hall’s panicgrass), *P*. *virgatum* (Switchgrass), *Setaria italica* (Foxtail millet), *Setaria viridis* (Green foxtail), *Sorghum bicolor* (Sorghum), *Z*. *mays* and *T*. *aestivum* (**Table 7**). For each of the marker, significant hits ranged from 9 to 62 for UGEP19, 1 to 6 for UGEP68, 3 to 89 for UGEP13 and 0 to 16 for UGEP90. The sequence search revealed 207 hits for UGEP19, followed by 180 hits for UGEP13 across grass genomes. The other markers, UGEP90 exhibited 65 hits and UGEP68 showed 32 hits. The genomes of *B*. *distachyon* and *P*. *virgatum* had maximum hits spread across chromosomes. Except UGEP90, which did not have a hit on the *B*. *distachyon* genome, all markers were identified on the chromosome of all species. Few sequences also showed multiple hits on the same chromosome (**S3 Table**). For instance, UGEP19 displayed maximum of 24 hits on chromosome Bd4 in the *B*. *distachyon* genome. Further, the search for annotated genes related to low P tolerance, could be putatively associated with 11 candidate genes in 6 grass species that were located in close proximity of the query sequences. Eleven candidate genes were located on the genomes of *O*. *sativa*, *B*. *distachyon*, *P*. *halli*, *P*. *virgatum*, *S*. *italica* and *T*. *aestivum*. Four candidate genes were identified on *B*. *distachyon* genome, followed by three on *O*. *sativa*, and one each on other genomes.

**Table 7:** Number of significant hits obtained during cross genome synteny search for orthologous sequences of the QTLs related to traits associated to P starvation tolerance in finger millet.

Common name	Species	No. of significant hits (E < 0.01)				Pi homeostasis genes
		*qLRDW*.*1*	*qLRDW*.*2*	*qHSDW*.*1*	*qHRL*.*1*	
		(UGEP19)	(UGEP68)	(UGEP13)	(UGEP90)	
Purple false brome	*Brachypodium distachyon*	62	1	4	0	4
Purple false brome	*B*. *stacei*	20	2	4	6	-
Rice	*Oryza sativa*	15	4	23	3	3
Hall's panicgrass	*Panicum hallii*	13	1	14	8	1
Switchgrass	*P*. *virgatum*	15	1	89	16	1
Foxtail millet	*Setaria italica*	15	5	3	6	1
Green foxtail	*S*. *viridis*	15	4	5	5	-
Sorghum	*Sorghum bicolor*	9	6	3	8	-
Corn	*Zea mays*	14	4	22	7	-
Wheat	*Triticum aestivum*	29	4	13	6	1
**Total**		**207**	**32**	**180**	**65**	**11**

Of the eleven candidate genes, seven genes were found associated with P-use efficiency traits, such as Pi homeostasis and P starvation tolerance (**Table 8**). The remaining genes were associated with morphological traits such as shoot and root growth under P_*suf*_. For the marker linked to the QTL *qHSDW*.*1*, significant candidate gene hits included the *cytochrome P450* gene (LOC_Os12g09790.1) which was located 5233.2 kb upstream from marker on Chr12 and *O*. *sativa inorganic PHosphate Transporter1*;*8* (*OsPHT1;8*) gene (LOC_Os10g30790.2) which was located 16005.7 kb downstream on Chr10 from the UGEP13 sequences in *O*. *sativa* genome. *Basic helix-loop-helix* (*bHLH*) transcription factor (TF) gene was found on two loci (Bradi1g28230.3 and Bradi4g29990.1) at 23449.2 kb upstream and 35621.2 kb downstream on the linkage groups Bd1 and Bd4 respectively from QTL *qLRDW*.*1* (UGEP19) in *B*. *distachyon* genome. Additionally, two *WRKY* TF genes were found in the same genome proximal to UGEP19 sequences at 13056.5 kb and 26426.4 kb upstream on the linkage group Bd1. The *Panicum* genomes contained *Ser/Thr kinase* genes downstream from UPEP19 sequences at 1123.3 kb and 48862.2 kb distances respectively on Chr06 of *P*. *hallii* and Chr06a of *P*. *virgatum* (Pahal.F00213.1 and Pavir.Fa02162.1). The score values were also higher with 248.3 and 223.1 in *P*. *hallii* and *P*. *virgatum* respectively for marker UGEP19 with very low E-values. From the QTL *qLRDW*.*2* related sequence of UGEP68, there was a *bHLH* TF gene located 33833.2 kb downstream at *O*. *sativa* Chr2. *WRKY* TF gene was also located 26336.2 kb downstream from *qLRDW*.*2* in Scaffold 2 in *S*. *italica*. The marker sequences of UGEP90 linked to the QTL *qHRL*.*1* were found proximal to the *pectin methylesterase inhibitor* (*PMEI*) (Traes_4DL_E3AE59EA9.2) gene located 17.1 kb upstream in the scaffold ta_iwgsc_4dl_v3_14404266 in *T*. *aestivum* (**Table 8**).

**Table 8:** The details of candidate genes from other cereals based on the *in silico* comparative genomics analysis for the finger millet QTLs associated with seedling stage P responses.

QTL	Trait	Species	EST length (bp)*	Chrom	Score	E-Value	Identity (%)	Transcript	Gene	Position	Function
*qHSDW*.*1*(UGEP13)	HSDW	*Oryza sativa* (Rice)	1260	Chr12	46.4	5.1E-3	84.8	LOC_Os12g09790.1	*Cytochrome P450*	5233.2 kb (US)	Shoot growth
				Chr10	42.8	6.2E-2	81.8	LOC_Os10g30790.2	*OsPHT1;8*	16005.7 kb (DS)	P homeostasis
*qLRDW*.*1* (UGEP19)	LRDW	*Brachypodium distachyon* (Purple false brome)	1164	Bd1	53.6	2.3E-5	81.8	Bradi1g28230.3	*bHLH* (TF)	23449.2 kb (US)	P starvation tolerance
				Bd4	46.4	3.4E-3	80.4	Bradi4g29990.1	*bHLH* (TF)	35621.2 kb (DS)	
				Bd1	48.2	9.7E-4	78.9	Bradi1g16120.4	*WRKY* (TF)	13056.5 kb (US)	
				Bd1	48.2	9.7E-4	78.9	Bradi1g30870.1	*WRKY* (TF)	26426.4 kb (US)	
		*Panicum hallii* (Hall’s panic grass)		Chr06	248.3	1.1E-63	77.1	Pahal.F00213.1	*Ser/Thr kinase*	1123.3 kb (DS)	Early root growth and development
		*P*. *virgatum* (Switch grass)		Chr06a	223.1	1.3E-55	76.2	Pavir.Fa02162.1	*Ser/Thr kinase*	48862.2 kb (DS)	
*qLRDW*.*2* (UGEP68)	LRDW	*Oryza sativa* (Rice)	1203	Chr2	44.6	1.7E-2	84.8	LOC_Os02g55250.1	*bHLH* (TF)	33833.2 kb (DS)	P starvation tolerance
		*Setaria italica* (Foxtail millet)		Scaffold 2	42.8	6.4E-2	71.0	Seita.2G175200.1	*WRKY* (TF)	26336.2 kb (DS)	
*qHRL*.*1* (UGEP90)	HRL	*Triticum aestivum* (Wheat)	1544	4DL	42.8	9.5E-2	78.9	Traes_4DL_E3AE59EA9.2	*PMEI*	17.1 kb (US)	Primary root growth

HSDW, shoot dry weight under P_*suf*_; LRDW, root dry weight under P_*def*_; HRL, root length under P_*suf*_; US, upstream; DS, downstream; *bHLH*, *basic helix-loop-helix*; TF, transcription factor; *PMEI*, *pectin methylesterase inhibitor*; *OsPHT1*;*8*, *O*. *sativa inorganic PHosphate Transporter 1*;*8*: Chrom, chromosome

*EST length used for BLAST analysis

## Discussion

Globally, the phosphatic fertilizer applied to agricultural soils in the year 2000 totalled 14.2 teragram per year, more than half of which was applied to cereal crops [78]. Although an additional manurial input of P approximating to 9.6 teragram per year collectively surpassed the 12.3 teragram of P per year removal through crop harvests, approximately 30% of the global cropland suffered P deficiency [79]. Developing P deficiency tolerant varieties therefore is critical to all crops, to make them resilient to future threats of P starvation [4, 80]. Therefore, the present study, first of its kind in finger millet, details low P stress responses of a small but diverse panel of cultivars, and identify QTLs for seedling stage genotype responses under P deficient and sufficient conditions. To control the seedling growth, we used perlite as the medium in the present study which was known to be an ideal material substrate to study the genotype responses [81]. Perlite is an inert volcanic glass, highly porous, light weight and sterile that can support high water retention and drainage along with proper aeration supporting healthy root growth and anchorage [82].

Information on low P response of small millets is scanty in literature. In a recent attempt, Ceasar et al. [59] made maiden effort in foxtail millet and standardised Pi concentration of 300 μM and 10 μM as ideal levels for P sufficiency and P deficiency respectively for plant growth under hydroponic and perlite system. We have used the same Pi concentrations in this study to emulate P sufficiency and P deficiency in our study. Further, genotype constitution used was wide enough, comprising of finger millet genotypes sourced from 18 countries worldwide, having an average genetic distance of 74% [57]. We have also chosen to measure phenotypic traits such as root and shoot biomass and root architecture traits that are proven to respond to low P conditions in other crop species [45, 83–86] to evaluate the P starvation response of finger millet genotypes.

### The phenotypic P response of genotypes

The phenotypic performance of genotypes showed distinct response under P_*suf*_ and P_*def*_ conditions, indicating variation in adaptive responses to P starvation. Although finger millet is a hardy crop capable of surviving under marginal conditions, the number of genotypes showing P starvation tolerance response can be expected to be high. By and large, under P_*def*_, the plants which responded positively to tolerate P starvation produced longer shoots, and induced several root hairs that were longer than those produced under P_*suf*_. However, root length showed a general trend of shortening among most of the genotypes which produced higher SL, RHD and RHL. This trend is similar to *O*. *sativa* genotypes exposed to low P condition wherein increase in RHL and RHD have been reported [87]. Root hairs are well known to play a major role in nutrient uptake especially in P acquisition under P_*def*_ [44, 88, 89], and also in root penetration in hard soil pans [90]. However, increased root length under P_*def*_ can be of additional benefit as it can drive nutrient foraging to wider and deeper areas [91].

### P deficiency stress inhibited biomass production

Significant reduction in shoot and root biomass was seen among the genotypes by 15^th^ day after germination in low P condition. Since biomass accumulation depends on P uptake, relative reduction in biomass can be expected under P deficiency. Root biomass has been recognised as one of the key traits for determining P starvation responses in *Z*. *mays* [92] and *G*. *max* [93]. In *O*. *sativa*, Wissuwa et al. [94] observed decrease in dry weight, tillering ability and P uptake to the tune of 50.4%, 46.7% and 61% respectively after 125 days of sowing among 98 backcross inbred lines under P starvation. In the present study, we have observed that the fresh weight of the samples had erratic variations, and hence we have limited the data to shoot and root dry weights. This can be attributed to experimental error rather than biological reasons, because the size of the plant samples handled was very small and therefore moisture level was beyond control in fresh samples [93]. Moisture and humidity are considered to affect correlation between fresh and dry weights in biological samples [95]. Considering this, among biomass traits, dry weight of the samples was more reliable than fresh weight. Other than the root and shoot biomass traits, relative tillering ability was also considered as an efficient parameter of P deficiency tolerance in *O*. *sativa* [96]. However, in the present work, tillering ability was not studied as the evaluation was confined to seedling stage. Since, there is no information available pertaining to P starvation response in finger millet, it may be interesting to observe tillering response under P starvation in future studies.

### Shoot length increased under P_*def*_ *vis-à-vis* P_*suf*_

The finger millet genotypes under P_*def*_ produced longer shoots than under P_*suf*_ until 30 days after germination. Further, the increased shoot length did not reflect in increased shoot weight under P_*def*_, indicating the possible role of cell elongation rather than cell multiplication in inducing the shoot length at early seedling stage in finger millet, corroborating tissue and cell type dependent plant response to P deficiency [4]. This observation was interesting, since reports from other grass species showed decline in shoot length under P_*def*_ condition. Cell production was found to be reduced by 19% and cell elongation by 20% in *Lolium perenne* after growing for 60 days under P deficiency [97]. In *O*. *sativa*, Luquet et al. [98] opined that under P deficiency, decrease in shoot growth gave advantage to the root system manifestation. It is well documented that plants preferentially allocate resources to increase below ground biomass and growth under P deficiency [99]. Even it has been proved in many plants that the resource allocation to below ground occurs during P deficiency at the expense of growth and photosynthesis [13, 100, 101]. In our study, root system manifestation under P_*def*_ treatment was primarily through root hair growth, while root length decreased. These observations provide new leads for further investigations of phenological behaviour in finger millet under P starvation.

### Root length under P_*def*_ condition

Plants under P starvation tend to modify their nutrient foraging behaviour through architectural manifestations of primary and lateral roots [86]. Major root traits that show modifications on low P stress are RL, lateral branching, branching angle, RHL and RHD [84, 102, 103]. In this study, RL behaviour under P_*def*_ treatment showed two distinct patterns among low P tolerant genotypes that showed positive responses with respect to traits such as SL, RHD and RHL. Among the 55 genotypes, 39 genotypes had reduced RL under P_*def*_ while 12 genotypes showed increased RL. Increased RL among P efficient genotypes clearly indicated adaptive response that would be useful for primary screening for P deficiency tolerance in finger millet. Accordingly, genotypes GPU45, IE5201, IE2871, IE7320, GPU66, HOSUR1, TCUM1, IE2034, SVK1, RAU8, VR708 and IE3391 can be considered as most efficient low P tolerant genotypes in this study, among which RAU8 and TCUM1 were having the longest roots under P_*def*_ treatment. In *Z*. *mays*, P efficient genotypes produced comparatively larger root system and showed higher total RL under P deficiency [104]. However, genotypes that had lower RL but low P response for other traits are also to be considered as good candidate varieties for P_*def*_ soils. In *O*. *sativa*, Wissuwa et al. [43] opined that the reduction of root growth under P deficiency was not affected by source limitations, but was due to a direct negative effect of P starvation on root growth.

### Induction of root hairs under P starvation

Most conspicuous observation in the present study was the enhanced root hair production under P_*def*_ treatment among several finger millet genotypes. Both RHD and RHL showed increased trend under P deficiency, indicating this to be a key adaptive behaviour against stress [105, 106]. The role of root hairs in significantly increasing P acquisition and utilization has been reported in *Hordeum vulgare* (Barley) [107], *O*. *sativa* [87] and *T*. *aestivum* [108]. Under P starvation, root hair production is triggered from production of trichoblasts by ectopic differentiation of root epidermal cells in root hair and non-root hair positions as well as by elongation suppression of root epidermal cells [85]. However, measurement of root hair parameters is a cumbersome process, especially under field grown conditions. There was no correlation between RHD and RHL in this study. In *G*. *max*, there was a negative correlation observed between the RHL and RHD which might be interpreted as a trade-off in terms of carbon use efficiency since combining both RHL and RHD would be too costly in terms of carbon usage [109]. But both higher RHL and RHD were found in P-efficient genotypes of *Phaseolus vulgaris* (Common bean) [54]. Wissuwa et al. [45] warned that leveraging of the measurement process could associate with errors of non-conformity since RHL and RHD varied tremendously between field-grown and hydroponic conditions [110].

### QTLs for P response traits

Selection of candidate traits is important for the identification of precise markers linked to the trait. AM is more precise than the linkage based mapping because it uses multitude of evolutionary recombination and strict filtering of false associations from an array of markers. Marker density depends on breeding behavior of the species; hence allogamous species require dense markers covering the entire genome while autogamous species require relatively less dense marker coverage [111, 112]. Being highly autogamous species, finger millet may harbor relatively larger haplotype blocks, thereby permitting to extend a specific marker association to a larger region of the genome (haplotype) for the candidate gene proximity [113]. Adhering to this principle, in the absence of whole genome information in finger millet and with few available genome wide markers, we proceeded with the only available microsatellite markers in this study. Since there are no reported marker-trait associations or candidate genes for low P response in finger millet, this study forms a maiden attempt to identify associations between random microsatellite markers and a set of candidate traits that are recognized as key players for low P responses [114] as exhibited by other crop species. The markers revealed the subtle population structure of the germplasm assembly by stratifying it into three subpopulations, making it an ideal panel for LD mapping. The decisive population structure provides a strong control in suppressing false associations [115]. However, due to the absence of information on genome location of the markers, exact LD pattern of the markers used could not be deciphered in this study. This can be assessed as soon as the whole genome information of finger millet is available, which can further boost the QTL discovery. In this direction, a very recent partial genome information of finger millet is published [116] which can be used for extensive investigations of the leads obtained from this study. The discovery of four markers associated with three traits linked to P responses, such as RDW under P_*def*_, and SDW and RL under P_*suf*_, showed stringency of declaring QTLs under MLM based AM. None of these markers have been assigned to finger millet linkage group so far [29, 117]. Moreover, in the absence of linkage map information, we are unable to conclude the proximity of the identified markers with previously reported markers in finger millet for blast resistance, agronomic traits and tryptophan content [31–33]. The QTLs are named as *qLRDW*.*1*, *qLRDW*.*2*, *qHSDW*.*1* and *qHRL*.*1* sans chromosome number following the international conventions as followed in *O*. *sativa* [118]. The present attempt also revealed that microsatellite markers are suitable for QTL mapping using AM approach in finger millet. Earlier, microsatellite markers were used for mapping QTLs for Pi efficiency related traits in *G*. *max* [51] and *O*. *sativa* [48] using linkage mapping approach. This study accounts for the first time report of the QTLs for P response traits in finger millet. This information can be an addition to the minimal number of QTLs for P-related traits so far reported in cereal crops [6].

### Identification of putative candidate genes for P response

Exploitation of cross-genome synteny between related genera is a powerful tool in comparative genomics for analyzing conserved regions across genomes and for identifying genes that share common functions [119, 120]. After the release of foxtail millet genome [121, 122], decoding finger millet genome is underway with the initiative from BioInnovate Africa (Bio-resources Innovation Network for Eastern Africa Development) with the partnership from African Orphan Crop Consortium and coordinated by ICRISAT regional team in Kenya (www.bioinnovate-africa.org) and is expected to be announced soon. In this context, we have used candidate gene tracking for the QTLs identified. In this study the length of the query sequences varied from 1164 bp (UGEP19) to 1544 bp (UGEP90), and the contig size was sufficient enough to make significant hits on reference genomes. The hits were explored for annotated gene sequences that have known functions related to the P responses identified in the study. The candidate genes reported here remain putative, pending validation. The present study was different from candidate gene based AM reported earlier in finger millet [32, 33, 123], as it could identify non-target candidate genes that have not been included in the candidate gene approach. Similar technique of cross-genome synteny was reported in finger millet for blast tolerance [34].

A total of 484 hits for the query sequences on ten reference grass genomes indicated high level of cross genome synteny with finger millet genome sequences. It has been reported that grass genomes are highly syntenous [124] and the major differences can be attributed to repetitive DNA sequences [125]. In *O*. *sativa*, *OsPSTOL1*, the major gene responsible for P starvation tolerance, is identified as a *Ser/Thr protein kinase*, the key gene for the *O*. *sativa* QTL *Pup1* [38]. *OsPSTOL1* is known to enhance crown root growth in the early root growth and development stages in *O*. *sativa* under P deficiency, enabling greater nutrient uptake by increasing root surface area [12]. Therefore, seedling stage expression of *Ser-Thr Kinase* under low P may be considered as a conducive strategy for P starvation tolerance in finger millet. In *O*. *sativa*, *Pup1* QTL has been successfully employed in marker assisted improvement of P starvation tolerance [126]. A biochemical pathway search (**Table 9**) of *Ser-Thr protein kinase* revealed that this enzyme forms a part of a signaling cascade of mitogen activated protein kinases (MAPK) that are involved in root growth and development [127].

**Table 9:** The details of biochemical pathways of putative candidate genes linked to the P starvation response in finger millet, based on the KEGG pathway database search.

Candidate gene	KEGG pathway	Pathway ID	Function/Description	Gene ID	References
*Cytochrome P450*	Brassinosteroid biosynthesis	Osa00905	Growth and development	4332134	[128]
*OsPHT1;8*	NA	NA	Phosphate; H^+^ symporter	4331542	[129]
*bHLH* (TF)	NA	NA	P starvation tolerance	*	[130]
*WRKY* (TF)	MAPK signaling pathway	Osa04016	P starvation tolerance	*	[131]
*Ser/Thr kinase protein*	MAPK signaling pathway	Osa04016	Root growth and development	*	[127]
*PMEI*	Pentose and glucoronate interconversion	Osa00040	Carbohydrate metabolism	4345722	[132, 133]

NA, not available

*, several members available

Other genes identified for low P response in the present study were shared between two families of TF, *bHLH* and *WRKY* and associated with P starvation tolerance. They act as suitable regulators for low P related gene cascade when challenged with low P conditions and play a fundamental role in P starvation tolerance [5]. Reports indicate that during P starvation, *bHLH* is downregulated and *WRKY* is upregulated and they have been identified to be linked with the alterations of root architecture [130, 134]. Both these group of genes were identified in *B*. *distachyon*, *O*. *sativa* and *S*. *italica* in our study. In *O*. *sativa*, 167 *bHLH* TF involved in a variety of functions has been identified genome wide [135]. *OsPTF1* responsible for imparting low P tolerance identified on chromosome 6 is a *bHLH* TF [136]. The *bHLH* TF is also known to play a role in root hair development as a response to low P stress [85]. Similar to *bHLH* family of TF, *WRKY* genes are also implicated in P acquisition in several studies. *WRKY75* was reported as one of the key regulator of P starvation response in *Arabidopsis* [134]. Recently, *OsWRKY74*, a member of group III *WRKY* TF family was demonstrated to be involved in P starvation tolerance in *O*. *sativa* [137]. Pathway analysis for these TFs using KEGG database, indicated several complex roles, such as *WRKY* involved predominantly acting in signaling pathways such as MAPK, leading to P starvation tolerance [131].

Additionally, we have identified three genes involved in phenotype response under P_*suf*_ such as the *Cytochrome P450*, *OsPHT1*;*8* and *PMEI* gene. *Cytochrome P450* is recognized as a gene involved in plant growth in *O*. *sativa* [138]. *OsPHT1*;*8* is an important phosphate transporter in *O*. *sativa*. Expression of *GUS* and *GFP* reporter genes driven by *OsPHT1*;*8* promoter showed that *OsPHT1;8* is expressed in various tissue organs from roots to seeds independent of Pi supply [129]. When expressed in *Xenopus oocytes*, it exhibited a *Km* of 23 μM confirming high affinity nature of this transporter. Knockdown of *OsPHT1*;8 by RNA interference decreased Pi uptake and plant growth under both high and low Pi conditions [129]. Recent study has also confirmed its role in Pi homeostasis especially in the movement of Pi from source to sink organs and allocation between embryo and endosperm of seeds [139]. Furthermore, *PMEI* genes are pivotal for cell wall formation in plants [133] and are reported to be essential for primary growth in *T*. *aestivum* [140]; these three genes were associated with high phosphate traits. Pathway search for these genes indicated that cytochrome P450 is involved in brassinosteroid biosynthesis which plays a crucial role in plant growth and development [128]. Similarly, PMEI plays an active role in pentose and glucuronate interconversions which is involved in carbohydrate metabolism [132, 133]. Although all genes reported in this study need individual validation for their mechanism and function in finger millet, plethora of supporting evidences from other crops and related species indicate their possible role in imparting P response, importantly P starvation tolerance. Once having validated, the QTLs can be directly used in MAS for breeding; however, information on the underlying genes can increase selection accuracy by developing a gene-based MAS system [141].

## Conclusion

This paper reports for the first time, mapping of QTLs for seedling stage low P stress response in finger millet, using germplasms of diverse geographical origin distributed worldwide. We have shortlisted ten low P tolerant genotypes that showed overall better performance for all the traits under investigation. Four QTLs were identified, of which two were linked to low P response, and putatively associated to eight candidate genes. One of the key leads towards the potential low P response gene was observed with the perceptible role of a *Ser/Thr protein kinase* gene in controlling root architecture. Since P deficiency at earlier stage in crop phenology may be detrimental to crop growth and establishment, seedling stage P deficiency tolerance is an essential trait that needs to be present in future climate resilient finger millet cultivars. Our results provide opportunity to breed low P tolerant finger millet genotypes in future using MAS. Further, our data throw light on several future leads on complex response of low P tolerance that needs detailed investigations such as the mechanisms in different finger millet genotypes. The selected germplasm lines can be used either as cultivars for marginal lands where P deficiency is prominent as well as donors for P starvation tolerance QTLs in future breeding.

## Supporting information

S1 Table The mean values of seedling stage P response in finger millet genotypes. (PDF)

S2 Table Genotypes matrix showing low P tolerant responses for different combinations of traits in which shorter root length under P_*def*_ was used as low P response. (PDF)

S3 Table The details of hits obtained with original sequences of QTLs UGEP13, UGEP19, UGEP68 and UGEP90 during cross genome synteny of ten species from grass family such as *Oryza sativa*, *Brachypodium distachyon*, *B*. *stacei*, *Panicum hallii*, *P*. *virgatum*, *Setaria italica*, *Setaria viridis*, *Sorghum bicolor*, *Zea mays* and *Triticum aestivum*. (PDF)
