# Supplementary material for: Identification of putative QTLs for seedling stage phosphorus starvation response in finger millet (Eleusine coracana L. Gaertn.) by association mapping and cross species synteny analysis
Source: PLoS One. 2017 Aug 18;12(8):e0183261. doi: 10.1371/journal.pone.0183261 (PMC5562303; doi:10.1371/journal.pone.0183261)
Supplement: S1 Table — (PDF) [file pone.0183261.s001.pdf]

S1 Table. The mean values of seedling stage P response in finger millet genotypes

Genotypes	SDW		RDW		SL		RL		RHD		RHL	
	P_{suf}	P_{def}	P_{suf}	P_{def}	P_{suf}	P_{def}	P_{suf}	P_{def}	P_{suf}	P_{def}	P_{suf}	P_{def}
APSSK1	2.9	3.5	1.5	1.7	9.3	7.9	7.3	7.8	38.7	44.3	8.7	9
CO11	4.6	2.3	2	1.6	7.8	5.3	6.4	8.3	20	30.3	4.7	7.3
CO12	6	3.8	2	2.3	8.3	10.1	8.6	9.7	23.7	21	9	6.7
CO14	5	3.4	1.9	1.8	9.7	4.9	6.4	9.2	31.3	27	3.3	8.3
CO7	4.5	3.3	2.2	2.7	5.9	8	10.4	8	16.7	22.3	9	6.7
CO9	4.8	1.7	1.1	1.9	14.2	6.5	8.7	6.7	20	22.7	9.7	10
CONO1	2	2.3	1.4	2.3	6.7	6.1	8	5.7	34.7	38.3	9.3	8.3
DPI00904	4.8	4.1	1.3	1.9	7.7	7.6	11.4	6.8	10.7	31.3	7.3	7
GPU26	5.3	2.5	2.6	2	7.8	6.8	8.1	7.9	28	29.7	7.7	9.7
GPU28	4	2.5	1.9	1.9	3.6	6.7	6	7.3	33.7	29.3	5.7	10.3
GPU45	2.6	2.6	1.6	2.3	6.2	13.7	6.3	8.6	23.7	35.7	7.3	12.7
GPU46	2.4	2.6	2.1	2.1	5.8	12.6	7.2	8.2	27	32.7	9	10
GPU48	5	5.6	2.1	2.4	6.4	11.5	10.5	8.1	25.7	22	13.3	9.3
GPU66	1.6	2.3	1.5	1.9	5.4	5.9	6.7	8.9	22	27	6	10.3
GPU67	1.6	1.6	1.3	1.8	5.7	7	7.2	6.6	26.3	36.7	11	8.7
HOSUR1	2.4	4.2	2	2	5.7	14.9	7.8	11.3	22.3	30.7	4.7	5.3
HR374	6.2	5.5	2.2	2.3	11.1	11.3	11.5	7.4	21	25.7	9.3	8.3
HR911	6.1	1.9	2.3	1.8	6.3	4.7	9.6	7	15	18	5.3	8.7
IE1055	3.7	2.9	1.3	1.6	4.8	6.6	7.8	7.1	25.7	21.3	10	9
IE2034	2.3	1.3	1.1	1.6	5.1	9.2	6.4	7.7	24	29.7	5.3	6.7
IE2042	1.2	1.3	1	1	5.2	5.9	7.4	5	17.7	25.7	4.7	10.3
IE2043	4.4	2.4	1.8	1.1	5.1	7	8.2	6	27.7	30.3	6.7	10.6
IE2217	5.6	2.2	2.5	1.4	8.1	6.5	8.2	7	21.7	33	5.7	9.7
IE2296	5.2	2.2	2.2	0.9	8.5	7.1	8.2	7.8	34	29.3	6.3	7.7
IE2312	4	2.7	3	1.4	16.7	6.6	15.8	6.2	19.7	15.3	3	6.7
IE2430	2.2	2.2	1.2	1.1	5.3	4.5	8.9	8.1	10	28.3	10.7	12
IE2437	4.4	3.7	1.5	1.2	3.2	7.7	10.1	7.1	21	21.3	7	11
IE2457	4.3	2.2	1.5	1.5	3.6	6	10.2	8.1	21.7	25.7	8.3	12
IE2572	5.2	4	2.2	1.9	4.2	5.4	11.5	6.5	20.3	28.3	5.7	9.7
IE2589	5.4	3.2	2.5	1.9	5.5	7.1	10.6	5.6	23	24.3	5	8.7
IE2606	15.3	2.6	4.2	1.5	11	8.2	9.5	8	10.3	27.7	7	9.3
IE2619	3	4.2	1.9	1.7	12.1	5.7	15.3	8.7	17.7	27.3	3.7	10.7
IE2710	5	2.6	1.8	1.6	5.5	8.1	10.6	9.2	37.3	24.7	10	8.3
IE2790	4.5	3	1.3	1	4.8	9.2	11.2	5.5	35	22.7	4.7	10
IE2821	4.4	3.2	2.3	1.8	9	6.1	11.8	9	27.7	23.3	14.7	10.3
IE2871	1.7	2.3	1.3	2.1	2.5	8	7	8.2	16	24	8	12.7
IE2872	7.7	6.4	2.1	1.9	5.3	7.5	11.9	8.1	29	25.7	4.7	11
IE2911	3.4	7	1.4	2.4	6.7	9.4	11.8	8.3	32.7	32.3	9.3	11
IE2957	3.5	4.8	2.6	2.3	4.2	6.2	11.5	8.2	14.7	26.3	9.3	12
IE3045	4.9	2.5	2.3	1.7	7.6	8.3	11.1	5.4	27.3	29.7	7	10.3
IE3077	3.7	5.1	2.6	2.6	5.8	10	11.5	8	16.7	24.3	11.3	8
IE3104	5.3	8.3	1.3	4	5.2	14.4	10.4	11.1	25.3	33	7.3	4
IE3317	3.9	2.6	1.8	1.4	7.6	6.1	9.1	8.8	36	24.3	5.7	7.7
IE3391	3.7	2.6	1	1	4.3	5.6	9.1	11.8	22	25	6.3	10

Genotypes	SDW		RDW		SL		RL		RHD		RHL	
	P_{suf}	P_{def}	P_{suf}	P_{def}	P_{suf}	P_{def}	P_{suf}	P_{def}	P_{suf}	P_{def}	P_{suf}	P_{def}
IE3392	3.7	2.2	1.1	2.4	6.1	7.6	9.5	6.2	21	26.3	7.3	9
IE3470	4.4	3.2	1.6	1.3	4.9	5.8	11.1	6.1	23	33	4.3	8.7
IE3475	4.7	2.5	1.5	0.9	5.9	6.8	8.1	10.5	21	21	8.7	5.3
IE3614	3.4	3.3	1.4	1.4	5.8	5.9	7.9	7.9	23.7	32.7	11	8.3
IE3618	4.5	2.5	0.7	0.9	7.5	5.4	4.8	6.6	20.3	36.3	6.7	8.3
IE3721	4.6	3.7	2.5	1.4	7.9	7.5	9	8.4	32.7	30	5.7	10.3
IE3945	2.7	3.1	2.1	2	3.4	5.6	4.1	7.8	26	32.3	11.3	6.3
IE3952	2.2	2.5	1.7	1.5	6.6	10	6.1	6.6	15.3	26.3	12.3	8
IE3973	3.9	2.4	1.1	1.2	10.3	6.9	8.4	5.8	19.3	20.7	6	7
IE4028	7.1	3.6	2.5	1.6	8.3	8.3	8.7	9.4	18.3	26.3	6.3	8.7
IE4057	5.8	4.8	1.2	1.7	5.3	7.5	13.8	10.6	14.7	26.3	6	8.7
IE4073	3.4	3.6	1.4	2.3	4.9	7.6	7.9	7.8	19.3	34.3	11	8.7
IE4121	4.1	3.6	1.4	1.5	5.9	6.3	11.9	7.1	33.7	30.3	9.7	12
IE4329	6.1	3.6	2.2	1.4	7.7	7.7	9.8	6.9	23	36.7	7	10.7
IE4491	5.9	2.7	2.7	1.5	6	8.6	13.8	9.5	20.7	46.3	6	11
IE4497	3.9	7.3	1.4	2.7	5.9	6.2	9.4	8.8	20	43.3	5.3	8.3
IE4545	4.1	6	1.4	1.6	3	8.5	12.3	7.9	20.7	25.3	3.7	10
IE4565	2.5	1	0.8	1.3	7.1	7.8	10.3	7.5	7.7	33.3	11.7	9.3
IE4570	3.5	3.3	1.4	1.2	5.9	15	10.4	7.6	27.3	37.7	5	11
IE4622	4.4	5.2	1.8	1.8	5.3	12.8	7.9	7.9	28.7	31.3	5.7	13
IE4646	4.4	3.3	1.9	2.4	8.6	8.3	6.1	10.9	28.7	40	10.3	12
IE4671	2.8	3.4	1.8	1.6	4	7	8.8	6.7	29.7	38	4.7	12
IE4673	3.4	3.2	1.5	1.3	6.7	6	10.3	9.9	22.3	31	5.7	11.7
IE4709	6.5	3.7	2.2	1.2	5.1	6.6	10.6	8.9	37	33.7	5.7	12.3
IE4734	11.7	6.4	4.1	1.9	8.8	8.7	9.6	8.3	27.7	31.7	12.7	12
IE4757	4.4	3.8	1.7	1.9	5.4	9.3	11	8.1	22.3	25.7	9.3	9.7
IE4795	2.8	5.4	1.3	2.1	6.8	6	16	10.7	25	37.7	2.3	12.7
IE4797	7.4	3.3	3.5	1.4	5.2	8.8	9.3	7.3	31.7	32.7	4.3	11
IE4816	2.9	3.7	1.6	1.2	5.4	8.1	12.3	8.5	39.7	39.3	4.7	10.3
IE501	4.7	3.5	2.1	0.9	5.3	7.6	9.9	6.5	21.7	24	9.7	11.3
IE5066	4.5	4.4	2.3	1.8	8.3	10.3	8.9	8.7	28.7	45.7	7.3	9.7
IE5091	3.2	4.6	1.8	2.2	5	7.2	12.1	7.8	33.7	32	7	12
IE5106	6.7	7.1	3.9	4.2	5.6	9.2	11.8	8.5	26.3	38.3	8.3	10.3
IE518	5.7	3.6	2.6	1.8	10.2	6.4	10.5	6.7	23.3	34.3	5	10.7
IE5201	4.1	2.7	2.3	0.8	6.4	9.4	6.6	8.1	29	40	9	10.7
IE5306	3.4	2.8	1.8	1.1	4.6	5.7	8.1	8	13.3	31	7	9.3
IE5367	3.5	4.1	1.6	1.6	5.3	9	9.3	7.9	29.7	31.7	8	11
IE5537	3.9	5.6	1.8	1.5	14.3	9.4	12.8	5.8	11.3	23.3	3	10
IE5817	6.3	4.6	2.1	1.5	6.4	8.2	12.2	6.4	13.3	26.3	5.3	7.7
IE5870	5.8	2.9	2	1.5	5	15.3	11.8	9.1	24.7	34	7	9.7
IE6059	5.3	2.4	2.5	1.3	8.1	7.5	9.7	7.7	25.7	27.3	7	11.3
IE6082	4.9	4.4	1.6	1.5	5.5	7.5	9.3	6.2	10	44.7	6	11.7
IE6154	3.5	2.7	1.1	2.2	5.7	6.4	9.8	7.4	24.3	29	9.3	7.3
IE6165	4.3	3.1	1.5	1.5	6.4	4.7	10.4	6.2	22.7	37.7	7.3	7
IE6221	3.9	2.1	1.3	1.4	5.6	5.1	8.6	11.7	14.7	38	5.3	10.7

Genotypes	SDW		RDW		SL		RL		RHD		RHL	
	P_{suf}	P_{def}	P_{suf}	P_{def}	P_{suf}	P_{def}	P_{suf}	P_{def}	P_{suf}	P_{def}	P_{suf}	P_{def}
IE6240	4.8	3.8	1.9	1.5	6	7	9.5	9.9	28	31	4.7	8
IE6294	5	3	1.5	0.9	7.2	8.9	10.6	7	14.7	31.7	11.7	9.3
IE6326	5	2.3	1.1	0.7	6	11.5	8.2	9.4	14	31.7	8.3	7.3
IE6337	2.9	4	1.7	1.3	5.5	7.5	9.3	10.9	34.3	34.3	8.7	9.7
IE6350	5.3	6	2.2	3	5.7	22.2	13.2	10.6	23.7	31.7	8	9.3
IE6421	10	4.7	4	1.6	11.1	6.4	9.7	8.1	31	43.3	8.7	6.3
IE6473	10.6	1.5	3.7	1.7	16.3	9.3	18.4	8.6	32	33	6.3	9.3
IE6514	2.9	2.9	1.3	1.7	7.3	11.3	10.8	7.6	25.7	40	7.3	9.3
IE6537	8.4	4.1	3.3	1.9	9	12.2	10.5	7.1	35.7	27.7	9.3	9.3
IE7018	11	3.9	4.4	1.1	11.2	9.1	9.8	8.3	36.3	38.3	8.7	11.3
IE7079	6	5	1.7	2.5	6.1	9.6	10.7	11	33.7	32.3	9	9.7
IE7320	3.5	7.7	1.9	2.4	7.5	14	9.7	11.8	28.3	36.3	5.3	14.7
INDOF5	2.5	1.7	1.5	1.4	5.9	7.3	9.2	9.2	14	32.3	9.7	10.3
INDOF7	2.2	2.6	1.2	1.3	6.3	7.9	9.4	9.6	35.3	36	5.7	11.3
INDOF8	3.3	2.7	1.5	2.1	5.7	5.1	12.1	11.5	20.7	26.7	3	10.3
INDOF9	4.6	7	1.8	2.9	6.3	14.9	12	11	15	34.7	8	11
KM252	4.3	2.4	2.3	1.3	5.6	7.5	8.4	7	22.3	30	12.7	13.3
KMR301	7.6	2.3	2.8	2	5.5	5.4	12.8	7.3	25.7	35.7	4.3	12
KRI00701	4	3.5	2.2	1.2	5.2	9.1	10	3.5	19	30.7	6.7	9.7
KRI1311	1.6	1.8	1.4	1.2	6.8	5.4	12.4	7.6	35	37.7	6.7	10.3
L5	4.3	4.9	2.6	1.3	4.6	10.2	8.5	7.7	13.7	45.3	4.7	7
ML365	4.9	4	3	2	5.4	8.5	9.8	6.8	13.3	25	9.3	11.3
MR1	4.8	3.9	1.7	1.7	4.6	11.2	9.1	6.9	23.3	32.3	9.7	9.7
MR2	14.4	4.4	5	2.4	21.4	7.1	21.5	7.7	26.3	35.7	5	10.3
MR6	6.4	3.9	2.6	1.4	7.4	8.4	12.9	9.8	21.7	48.7	4.7	8.7
PAIYUR2	2.8	3.4	1.7	1.8	4.3	10	12	7.3	13.3	29.7	9	10
PES110	3.7	4.2	2	1.2	5.2	13.4	12.9	7.4	23.7	25	10	7.7
PR202	3.6	4	2.1	2.6	4.9	7.8	12.4	9.1	22	31.3	9.7	13
RAU8	2.9	4.6	1.5	1.7	11.8	18.5	12.2	17.9	23	31.7	5.7	10
SVK1	1.6	1.2	1.1	1.1	5.3	6.1	5.4	6.4	28.7	40.3	8.3	10.3
TCHIN1	3.5	2.4	2.2	1.4	14	9.4	16.6	8.8	20.7	28.3	5.7	8
TCUM1	1.8	3.2	1.3	1.4	7.7	14.3	6.8	17.6	20.3	29.7	6	8.3
THRP1	3.5	2.6	1.7	1.5	6.4	5.9	7.2	5.8	19.3	32.3	8.3	8.7
THRVP	3.2	3.6	1.2	1.1	7.5	10	9.6	6.6	30.7	26.3	8.3	9.7
THRVPP	3	3	1.3	0.9	10.2	7.9	9.9	7.2	27.3	25.7	4	6.7
TRY1	2.7	3.7	1.4	1.4	4.8	8.2	8.8	7.7	16.7	25.7	8.7	10
VIJAYAWADA	3.6	3.2	2	2.2	3.9	8.4	11.1	6.6	18.3	25.3	8.7	9.3
VL149	2.8	3.6	1.3	2.1	5.6	8.9	6	5.6	13.3	43	5.3	12.7
VR708	3.5	4.4	1.4	2.2	6.6	8.7	7.5	8.8	22	27.7	7.3	8.3
Mean	4.5	3.5	1.9	1.7	6.8	8.4	9.9	8.1	23.7	31	7.4	9.6

The data was collected after 15 days of sowing for SDW and RDW and 30 days of sowing for SL, RL, RHD and RHL.
P_{def}, P-deficient conditions; P_{suf}, P-sufficient conditions
