# Supplementary material for: Identification of putative QTLs for seedling stage phosphorus starvation response in finger millet (Eleusine coracana L. Gaertn.) by association mapping and cross species synteny analysis
Source: PLoS One. 2017 Aug 18;12(8):e0183261. doi: 10.1371/journal.pone.0183261 (PMC5562303; doi:10.1371/journal.pone.0183261)
Supplement: S2 Table — (PDF) [file pone.0183261.s002.PDF]

S2 Table. Genotypes matrix showing low P tolerant responses for different combinations of traits in which shorter root length under P_{def} was used as low P response

Traits	Total	Genotypes
Root hair density<br>Root hair length<br>Root length<br>Shoot length	39	IE5106, IE2043, PR202, VL149, IE2589, ML365, IE4797, PAIYUR2, IE5367, MR6, IE4671, KM252, IE6082, IE2042, IE5870, INDOF9, IE3470, IE6350, IE4491, IE4570, IE3045, IE5066, IE4057, IE2457, VIJAYAWADA, IE6514, KRI00701, IE4622, IE2957, IE2437, IE4545, IE5306, IE5817, IE501, TRY1, L5, IE2572, IE4757, IE3392
Root hair density<br>Root length<br>Shoot length	9	PES110, IE4073, IE6294, IE6154, IE3077, MR1, GPU67, CO7, IE4565
Root hair length<br>Root length<br>Shoot length	8	IE4816, IE2911, IE2790, IE5091, IE4709, IE2872, THRVP, IE4121
Root hair density<br>Root hair length<br>Shoot length	16	GPU45, IE5201, IE2871, IE6240, IE7320, TCUM1, SVK1, VR708, IE3391, GPU46, GPU66, HOSUR1, IE2034, RAU8, INDOF7, INDOF5
Root hair density<br>Root hair length<br>Root length	22	MR2, IE4795, IE6473, IE5537, IE2217, IE3973, IE7018, GPU26, IE2606, IE2430, THRP1, KMR301, IE4497, IE518, INDOF8, TCHIN1, HR911, IE4673, IE2619, IE6059, IE4329, KRI1311
Root length<br>Shoot length	4	IE2710, IE6537, IE1055, GPU48
Root hair density<br>Shoot length	5	IE6326, IE3475, IE3952, IE3945, IE3104
Root hair length<br>Shoot length	3	GPU28, IE7079, IE6337
Root hair density<br>Root length	8	CONO1, IE3614, IE6165, DPI00904, IE4734, IE6421, HR374, CO9
Root hair length<br>Root length	5	IE2296, IE3721, THRVPP, IE2312, IE3317
Root hair density<br>Root hair length	5	IE6221, IE4646, CO11, IE4028, IE3618
Shoot length	1	CO12
Root length	1	IE2821
Root hair density	1	APSSK1
Root hair length	1	CO14

SL, shoot length; RL, root length; RHD, root hair density per 10 µm primary root length; RHL, root hair length
