# Supplementary material for: Identification of putative QTLs for seedling stage phosphorus starvation response in finger millet (Eleusine coracana L. Gaertn.) by association mapping and cross species synteny analysis
Source: PLoS One. 2017 Aug 18;12(8):e0183261. doi: 10.1371/journal.pone.0183261 (PMC5562303; doi:10.1371/journal.pone.0183261)
Supplement: S3 Table — (PDF) [file pone.0183261.s003.PDF]

S3 Table. The details of hits obtained with original sequences of QTLs UGEP13, UGEP19, UGEP68 and UGEP90 during cross genome synteny of ten species from grass family such as *Oryza sativa*, *Brachypodium distachyon*, *B. stacei*, *Panicum hallii*, *P. virgatum*, *Setaria italica*, *Setaria viridis*, *Sorghum bicolor*, *Zea mays* and *Triticum aestivum*

Marker: UGEP13

Species	Chromosome/ Linkage group	Identity	Start	End	E value	Score
Brachypodium distachyon	Bd3	82.35	35520420	35520370	9.00E-05	51.8
	Bd3	80.39	32406045	32405997	1.30E-02	44.6
	Bd5	80.77	20218818	20218869	3.00E-04	50
	Bd4	79.31	25927814	25927868	1.00E-03	48.2
Brachypodium stacei	Chr02	91.11	24174079	24174123	1.00E-08	64.4
	Chr10	83.02	96285	96233	2.00E-05	53.6
	Chr06	82.98	19828518	19828564	3.00E-04	50
	Chr09	82.98	5730642	5730688	1.00E-03	48.2
Oryza sativa	Chr8	92.59	15461193	15461242	9.00E-13	78.8
	Chr8	93.02	27756986	27756946	7.00E-08	62.6
	Chr8	100	22939174	22939200	4.00E-04	50
	Chr8	96.15	23081718	23081693	6.20E-02	42.8
	Chr1	83.08	1928123	1928179	3.00E-06	57.2
	Chr1	84.78	16097504	16097459	5.00E-03	46.4
	Chr1	87.88	10703638	10703606	6.20E-02	42.8
	Chr1	81.82	38729399	38729360	6.20E-02	42.8
	Chr2	80.7	6169436	6169380	3.00E-05	53.6
	Chr2	85.19	33846130	33846080	1.00E-04	51.8
	Chr2	78.57	6166166	6166111	1.00E-03	48.2
	Chr2	85.11	23488732	23488778	1.00E-03	48.2
	Chr9	82.35	4497847	4497891	1.00E-03	48.2
	Chr9	80.39	18620780	18620830	1.00E-03	48.2

Species	Chromosome/ Linkage group	Identity	Start	End	E value	Score
	Chr12	84.78	5223645	5223690	5.00E-03	46.4
	Chr6	84.78	20120927	20120972	5.00E-03	46.4
	Chr6	84.78	28532060	28532018	5.00E-03	46.4
	Chr6	81.4	25419476	25419434	6.20E-02	42.8
	Chr5	86.84	23180528	23180491	5.00E-03	46.4
	Chr4	83.33	8876755	8876714	1.80E-02	44.6
	Chr3	96.55	8984737	8984765	1.80E-02	44.6
	Chr3	88.24	15524710	15524677	1.80E-02	44.6
	Chr10	81.82	16005462	16005501	6.20E-02	42.8
Panicum hallii	Chr06	96	41902475	41902524	1.00E-13	82.4
	Chr03	94.12	2977353	2977303	1.00E-12	78.8
	Chr03	96.15	4125407	4125432	9.10E-02	42.8
	Chr03	92.86	46733305	46733332	9.10E-02	42.8
	Chr05	82	57306995	57306946	6.00E-04	50
	Chr05	86.36	40458037	40458080	9.10E-02	42.8
	Chr09	91.18	33142047	33142014	7.00E-03	46.4
	Chr09	85	709610	709649	9.10E-02	42.8
	Chr07	84.31	21191415	21191462	7.00E-03	46.4
	Chr07	86.49	31903735	31903771	2.60E-02	44.6
	Chr07	77.59	44347463	44347520	2.60E-02	44.6
	Chr07	90.32	31928268	31928298	9.10E-02	42.8
	Chr04	90.91	25266734	25266702	2.60E-02	44.6
	Chr01	93.94	47454460	47454428	2.60E-02	44.6
Panicum virgatum	contig77548	90.2	49	1	7.00E-09	68
	Chr01b	90.38	23745119	23745168	8.00E-08	64.4
	Chr01b	82.35	26356427	26356377	5.00E-04	51.8
	Chr01b	86.05	41196778	41196736	7.00E-03	48.2
	Chr01b	90.91	21266952	21266984	8.00E-02	44.6

Species	Chromosome/ Linkage group	Identity	Start	End	E value	Score
	Chr01b	90.91	21281916	21281948	8.00E-02	44.6
	Chr01b	96.3	29741451	29741425	8.00E-02	44.6
	Chr01b	83.72	38564105	38564147	8.00E-02	44.6
	Chr02b	81.97	24834970	24834912	4.00E-05	55.4
	Chr02b	81.82	15043959	15043897	5.00E-04	51.8
	Chr02b	84.21	18913109	18913056	7.00E-03	48.2
	Chr02b	90.91	39001015	39000983	8.00E-02	44.6
	Chr07b	82.26	13292825	13292765	2.00E-04	53.6
	Chr08b	82.35	48020488	48020438	5.00E-04	51.8
	Chr08b	84.21	26679703	26679652	2.30E-02	46.4
	contig379996	91.67	272	307	2.00E-03	50
	contig304775	91.67	1105	1140	2.00E-03	50
	Chr08a	78.12	829609	829670	2.00E-03	50
	Chr08a	93.94	27559353	27559321	7.00E-03	48.2
	Chr08a	80	29785039	29785098	2.30E-02	46.4
	Chr08a	88.89	31627545	31627510	8.00E-02	44.6
	Chr03b	82.14	14431281	14431334	2.00E-03	50
	Chr03b	93.94	16484614	16484646	7.00E-03	48.2
	Chr03b	86.05	32488678	32488720	7.00E-03	48.2
	Chr03b	96.3	28170373	28170347	8.00E-02	44.6
	Chr03b	93.1	35919628	35919600	8.00E-02	44.6
	Chr01a	84.31	11715187	11715237	2.00E-03	50
	Chr01a	90.91	54335680	54335648	8.00E-02	44.6
	Chr01a	91.18	56300365	56300332	8.00E-02	44.6
	contig378616	86.05	186	144	7.00E-03	48.2
	contig359741	86.05	54	96	7.00E-03	48.2
	contig316284	86.05	106	64	7.00E-03	48.2
	contig263601	86.05	1652	1610	7.00E-03	48.2

Species	Chromosome/ Linkage group	Identity	Start	End	E value	Score
	contig175862	86.05	2238	2280	7.00E-03	48.2
	contig168435	86.05	2383	2425	7.00E-03	48.2
	contig159363	86.05	900	942	7.00E-03	48.2
	contig131574	86.05	3017	3059	7.00E-03	48.2
	contig126657	86.05	297	255	7.00E-03	48.2
	contig122387	86.05	3184	3142	7.00E-03	48.2
	contig18861	93.94	6858	6890	7.00E-03	48.2
	contig07204	96.55	7209	7237	7.00E-03	48.2
	Chr09b	88.89	23553890	23553855	7.00E-03	48.2
	Chr09b	91.43	40068727	40068693	2.30E-02	46.4
	Chr09b	83.72	34085874	34085832	8.00E-02	44.6
	Chr09b	90.91	39247825	39247793	8.00E-02	44.6
	Chr09a	93.94	2650010	2649978	7.00E-03	48.2
	Chr09a	90.91	38644454	38644422	8.00E-02	44.6
	Chr09a	90.91	76448328	76448360	8.00E-02	44.6
	Chr06b	93.94	23979950	23979918	7.00E-03	48.2
	Chr06b	100	38925978	38925954	2.30E-02	46.4
	Chr06b	90.91	30352432	30352464	8.00E-02	44.6
	Chr05b	77.78	27519633	27519695	7.00E-03	48.2
	Chr05b	90.91	54364289	54364321	8.00E-02	44.6
	Chr04b	93.94	50940355	50940323	7.00E-03	48.2
	Chr04b	90.91	15318442	15318410	8.00E-02	44.6
	Chr04b	90.91	22751892	22751924	8.00E-02	44.6
	contig357797	91.43	47	13	2.30E-02	46.4
	contig355725	100	711	687	2.30E-02	46.4
	contig245789	80	1832	1880	2.30E-02	46.4
	contig142688	91.43	2337	2303	2.30E-02	46.4
	contig12167	91.43	1873	1839	2.30E-02	46.4

**Species**	**Chromosome/ Linkage group**	**Identity**	**Start**	**End**	**E value**	**Score**
	contig10815	81.13	2557	2505	2.30E-02	46.4
	Chr02a	91.67	48808438	48808404	2.30E-02	46.4
	Chr02a	88.89	35342020	35341985	8.00E-02	44.6
	Chr02a	91.18	35669757	35669790	8.00E-02	44.6
	Chr02a	83.72	36262325	36262283	8.00E-02	44.6
	contig408611	90.91	382	414	8.00E-02	44.6
	contig288025	90.91	694	726	8.00E-02	44.6
	contig274804	90.91	92	60	8.00E-02	44.6
	contig244606	90.91	870	838	8.00E-02	44.6
	contig238870	90.91	145	177	8.00E-02	44.6
	contig200164	77.78	419	366	8.00E-02	44.6
	contig175039	90.91	2164	2196	8.00E-02	44.6
	contig152136	83.33	245	201	8.00E-02	44.6
	contig137176	88.89	2948	2981	8.00E-02	44.6
	contig127088	90.91	671	703	8.00E-02	44.6
	contig126062	83.72	1106	1148	8.00E-02	44.6
	contig95420	83.72	59	17	8.00E-02	44.6
	contig81031	93.1	2437	2409	8.00E-02	44.6
	contig72841	90.91	4407	4375	8.00E-02	44.6
	contig61906	89.47	1587	1622	8.00E-02	44.6
	contig40302	82	4876	4925	8.00E-02	44.6
	contig05701	90.91	13048	13016	8.00E-02	44.6
	Chr06a	100	3959379	3959402	8.00E-02	44.6
	Chr06a	100	6528885	6528862	8.00E-02	44.6
	Chr04a	83.33	43259942	43259901	8.00E-02	44.6
	Chr03a	82	17383288	17383239	8.00E-02	44.6
	Chr03a	100	30725483	30725460	8.00E-02	44.6
	Chr03a	90.91	48066753	48066721	8.00E-02	44.6

Species	Chromosome/ Linkage group	Identity	Start	End	E value	Score
Setaria italica	scaffold_9	77.05	9502305	9502365	1.00E-05	55.4
	scaffold_9	77.42	20711928	20711983	5.00E-03	46.4
	scaffold_3	79.17	42906117	42906164	6.70E-02	42.8
Setaria viridis	Chr_09	88.33	52911996	52911937	9.00E-13	78.8
	Chr_09	77.05	9301959	9302019	1.00E-05	55.4
	Chr_09	80	36862110	36862051	4.00E-04	50
	Chr_03	80.77	8398373	8398418	5.00E-03	46.4
	Chr_02	92.86	14909888	14909915	6.50E-02	42.8
Sorghum bicolor	Chr06	87.5	30816073	30816036	3.00E-03	48.2
	Chr04	76.67	1403228	1403169	1.00E-02	46.4
	Chr01	82.69	17909151	17909200	1.00E-02	46.4
Zea mays	9	92.45	31030180	31030232	6.00E-11	75.2
	9	78.33	102842099	102842042	8.00E-03	48.2
	9	100	131127491	131127466	8.00E-03	48.2
	9	100	57879689	57879665	2.80E-02	46.4
	1	85.48	20687201	20687144	4.00E-06	59
	1	82.54	20609951	20610010	5.00E-05	55.4
	1	100	123324625	123324600	8.00E-03	48.2
	1	87.23	233985412	233985369	8.00E-03	48.2
	1	100	26088691	26088667	2.80E-02	46.4
	1	82.69	26231875	26231924	2.80E-02	46.4
	1	84.44	150008420	150008463	2.80E-02	46.4
	1	100	260093853	260093829	2.80E-02	46.4
	1	79.59	108343077	108343125	9.70E-02	44.6
	1	100	260138217	260138194	9.70E-02	44.6
	2	80.7	87563754	87563698	2.00E-04	53.6
	2	100	180721169	180721146	9.70E-02	44.6
	6	82.35	132235016	132235066	7.00E-04	51.8

Species	Chromosome/ Linkage group	Identity	Start	End	E value	Score
	6	81.03	67526218	67526274	8.00E-03	48.2
	4	100	235172620	235172646	2.00E-03	50
	4	86.05	119079653	119079611	8.00E-03	48.2
	5	82	144499352	144499303	2.00E-03	50
	7	82.98	46072752	46072707	2.80E-02	46.4
Triticum aestivum	ta_iwgsc_5dl_v1_4584636	94.12	10637	10687	1.00E-12	78.8
	ta_iwgsc_4bs_v1_4901407	89.29	218	273	2.00E-11	75.2
	ta_iwgsc_2dl_v1_9885701	90.2	2390	2440	7.00E-10	69.8
	ta_iwgsc_7dl_v1_3395182	88.24	3876	3826	9.00E-09	66.2
	ta_iwgsc_6dl_v1_3271976	84.91	2098	2150	5.00E-06	57.2
	ta_iwgsc_5dl_v1_4574152	81.67	14456	14397	5.00E-06	57.2
	ta_iwgsc_1bl_v1_3874034	86.96	308	263	5.00E-06	57.2
	ta_iwgsc_5bs_v1_2285955	82.61	2940	2985	2.00E-03	48.2
	ta_iwgsc_2bl_v1_8090779	89.19	23566	23532	9.00E-03	46.4
	ta_iwgsc_1bs_v1_3441641	79.25	11497	11445	9.00E-03	46.4
	ta_iwgsc_6as_v1_4412851	87.5	462	424	3.00E-02	44.6
	ta_iwgsc_2bl_v1_7970262	83.33	14107	14066	3.00E-02	44.6
	ta_iwgsc_1ds_v1_1898084	93.55	13328	13299	3.00E-02	44.6

Marker: UGEP19

Species	Chromosome / Linkage group	Identity	Start	End	E value	Score
Brachypodium distachyon	Bd3	68.28	11823962	11823674	9.00E-17	91.5
	Bd3	82.14	5850514	5850565	7.00E-06	55.4
	Bd3	81.13	12895890	12895938	3.00E-04	50
	Bd3	80.36	13097205	13097154	3.00E-04	50
	Bd3	80.36	16197522	16197573	3.00E-04	50
	Bd3	81.13	19996340	19996292	3.00E-04	50
	Bd3	84.44	42025301	42025341	3.00E-04	50

Species	Chromosome / Linkage group	Identity	Start	End	E value	Score
	Bd3	80.39	10116659	10116613	3.00E-03	46.4
	Bd3	81.25	14426612	14426565	3.00E-03	46.4
	Bd3	80.39	23278664	23278710	3.00E-03	46.4
	Bd3	78.57	49934810	49934861	3.00E-03	46.4
	Bd5	87.93	11563060	11563117	2.00E-07	60.8
	Bd5	87.23	11563059	11563101	2.00E-06	57.2
	Bd5	81.03	8561118	8561171	8.00E-05	51.8
	Bd5	81.13	5698389	5698437	3.00E-04	50
	Bd5	80.77	11056634	11056681	1.00E-03	48.2
	Bd5	80.39	9522031	9521985	3.00E-03	46.4
	Bd5	80.39	9998348	9998394	3.00E-03	46.4
	Bd4	87.04	24206239	24206186	5.00E-07	59
	Bd4	82.46	2299684	2299632	2.00E-06	57.2
	Bd4	81.82	11679243	11679293	2.00E-05	53.6
	Bd4	81.82	24206270	24206220	2.00E-05	53.6
	Bd4	85.19	28129610	28129558	2.00E-05	53.6
	Bd4	83.67	7983630	7983586	8.00E-05	51.8
	Bd4	79.37	23729103	23729161	8.00E-05	51.8
	Bd4	79.03	28129629	28129572	8.00E-05	51.8
	Bd4	81.03	32863146	32863093	8.00E-05	51.8
	Bd4	81.13	10399430	10399382	3.00E-04	50
	Bd4	81.13	14735114	14735066	3.00E-04	50
	Bd4	81.13	20159866	20159814	3.00E-04	50
	Bd4	80.3	24206271	24206206	3.00E-04	50
	Bd4	81.13	27219009	27218961	3.00E-04	50
	Bd4	84.44	34627389	34627349	3.00E-04	50
	Bd4	76.92	9344095	9344035	1.00E-03	48.2
	Bd4	78.33	19011164	19011109	1.00E-03	48.2
	Bd4	80	22754866	22754916	1.00E-03	48.2
	Bd4	80.39	9521480	9521434	3.00E-03	46.4
	Bd4	78.57	14866200	14866251	3.00E-03	46.4

**Species**	**Chromosome / Linkage group**	**Identity**	**Start**	**End**	**E value**	**Score**
	Bd4	78.57	28172776	28172725	3.00E-03	46.4
	Bd4	81.63	29035635	29035683	3.00E-03	46.4
	Bd4	80.39	35607179	35607225	3.00E-03	46.4
	Bd4	79.25	18896212	18896164	1.20E-02	44.6
	Bd1	82.76	54722731	54722674	2.00E-06	57.2
	Bd1	82.14	21454086	21454035	7.00E-06	55.4
	Bd1	81.82	23449348	23449398	2.00E-05	53.6
	Bd1	81.03	54717538	54717591	2.00E-05	53.6
	Bd1	81.03	43177432	43177485	8.00E-05	51.8
	Bd1	83.33	64483317	64483266	8.00E-05	51.8
	Bd1	81.13	3493364	3493316	3.00E-04	50
	Bd1	81.13	23449320	23449368	3.00E-04	50
	Bd1	81.13	54132895	54132943	3.00E-04	50
	Bd1	78.95	13056855	13056803	1.00E-03	48.2
	Bd1	78.95	26426759	26426707	1.00E-03	48.2
	Bd1	80	29242160	29242210	1.00E-03	48.2
	Bd1	80	31437485	31437535	1.00E-03	48.2
	Bd1	80.39	28521480	28521434	3.00E-03	46.4
	Bd1	80.39	43128314	43128360	3.00E-03	46.4
	Bd2	80.33	4816270	4816326	7.00E-06	55.4
	Bd2	83.67	4816252	4816296	8.00E-05	51.8
	Bd2	83.67	13226345	13226301	8.00E-05	51.8
	Bd2	81.13	35185517	35185565	3.00E-04	50
	Bd2	82.98	11374471	11374513	1.00E-03	48.2
Brachypodium stacei	Chr03	69.75	3188489	3188701	2.00E-17	93.3
	Chr03	80	10687328	10687278	8.00E-04	48.2
	Chr08	84.62	12314315	12314268	2.00E-06	57.2
	Chr08	83.02	12258688	12258738	7.00E-05	51.8
	Chr05	90.7	7756464	7756504	2.00E-06	57.2
	Chr05	80.7	7048605	7048551	7.00E-05	51.8
	Chr10	82.35	15642616	15642574	6.00E-06	55.4

Species	Chromosome / Linkage group	Identity	Start	End	E value	Score
	Chr06	85.11	6479671	6479625	2.00E-05	53.6
	Chr06	83.33	9718079	9718120	3.60E-02	42.8
	Chr02	83.33	17544595	17544644	2.00E-05	53.6
	Chr02	87.18	17544594	17544630	3.00E-03	46.4
	Chr09	80.7	9293146	9293200	7.00E-05	51.8
	Chr09	87.8	9151778	9151740	2.00E-04	50
	Chr09	77.59	4808709	4808652	3.00E-03	46.4
	Chr09	77.42	9293127	9293184	1.00E-02	44.6
	Chr04	87.8	17324397	17324435	2.00E-04	50
	Chr04	90.32	5152540	5152510	1.00E-02	44.6
	Chr07	82.22	12136454	12136414	1.00E-02	44.6
	Chr07	86.49	1046503	1046469	3.60E-02	42.8
	Chr01	82.22	17286841	17286799	3.60E-02	42.8
Oryza sativa	Chr8	73.08	426856	426519	9.00E-44	181
	Chr8	86.84	427889	427852	5.00E-03	46.4
	Chr8	77.59	426929	426880	1.60E-02	44.6
	Chr4	90.16	27042609	27042549	3.00E-12	77
	Chr4	88.89	31164142	31164182	9.00E-06	55.4
	Chr4	90.24	34730833	34730869	9.00E-06	55.4
	Chr4	90	4954280	4954309	5.70E-02	42.8
	Chr4	88.57	28374484	28374451	5.70E-02	42.8
	Chr1	90.38	36934652	36934703	1.00E-10	71.6
	Chr10	84.38	13292405	13292352	5.00E-09	66.2
	Chr10	82.46	14723172	14723218	7.00E-07	59
	Chr12	84.91	25289930	25289882	7.00E-07	59
	Chr3	82.69	24835229	24835276	1.00E-03	48.2
	Chr5	100	22789910	22789932	5.70E-02	42.8
	Chr2	90	22192929	22192958	5.70E-02	42.8
Panicum hallii	Chr06	77.05	1073396	1073067	1.00E-63	248
	Chr06	87.27	1103158	1103206	3.00E-08	64.4
	Chr06	82.98	1073501	1073457	7.00E-03	46.4

Species	Chromosome / Linkage group	Identity	Start	End	E value	Score
	Chr06	82.61	1587492	1587451	7.00E-03	46.4
	Chr07	83.33	32225949	32225842	2.00E-22	111
	Chr07	83.72	32226050	32226010	2.40E-02	44.6
	Chr03	89.29	4464532	4464583	5.00E-11	73.4
	Chr03	82.61	6720700	6720663	6.00E-04	50
	Chr08	85.19	2980306	2980351	3.00E-08	64.4
	Chr05	83.93	8488153	8488204	3.00E-07	60.8
	Chr05	84.62	54213979	54213936	3.00E-07	60.8
	Chr04	84.48	17702339	17702390	6.00E-04	50
	Chr09	91.89	70876361	70876397	7.00E-03	46.4
Panicum virgatum	contig182248	75.45	8	372	3.00E-63	248
	Chr06a	76.22	48811529	48811205	1.00E-55	223
	Chr06a	81.93	48812530	48812451	4.00E-12	78.8
	Chr06a	85.19	48811607	48811556	3.00E-06	59
	contig316264	82.5	136	20	3.00E-20	105
	Chr06b	82.5	373319	373203	3.00E-20	105
	Chr06b	81.93	374163	374084	4.00E-12	78.8
	Chr06b	87.18	3121334	3121300	6.00E-03	48.2
	Chr03b	81.93	10600043	10599964	4.00E-12	78.8
	Chr09b	100	9128185	9128221	6.00E-09	68
	Chr05b	90.74	4277276	4277329	6.00E-09	68
	Chr05b	81.4	10047963	10047929	7.30E-02	44.6
	contig19148	79.37	5751	5807	5.00E-04	51.8
	contig37323	81.82	1482	1447	2.10E-02	46.4
	Chr02a	81.13	58714204	58714252	2.10E-02	46.4
Setaria italica	scaffold_6	80.99	564552	564214	5.00E-85	318
	scaffold_6	83.33	565474	565395	2.00E-13	80.6
	scaffold_7	91.53	27314050	27313992	3.00E-12	77
	scaffold_7	85.45	22607878	22607924	5.00E-09	66.2
	scaffold_7	83.33	18296190	18296235	2.00E-07	60.8
	scaffold_5	91.38	7444323	7444266	1.00E-11	75.2

Species	Chromosome / Linkage group	Identity	Start	End	E value	Score
	scaffold_5	84.78	29005925	29005966	1.00E-04	51.8
	scaffold_5	82.14	31993892	31993947	1.00E-04	51.8
	scaffold_5	80	25643601	25643556	1.80E-02	44.6
	scaffold_2	91.38	5103718	5103661	1.00E-11	75.2
	scaffold_8	86.54	24030893	24030846	7.00E-08	62.6
	scaffold_1	83.33	28741007	28740958	3.00E-05	53.6
	scaffold_1	81.48	28740971	28740918	4.00E-04	50
	scaffold_1	77.55	8559201	8559241	6.20E-02	42.8
	scaffold_9	91.89	57661220	57661256	5.00E-03	46.4
Setaria viridis	Chr_06	80.99	541528	541190	5.00E-85	318
	Chr_06	83.33	542490	542411	2.00E-13	80.6
	Chr_07	91.53	26371096	26371038	3.00E-12	77
	Chr_07	91.38	24074145	24074088	1.00E-11	75.2
	Chr_07	89.29	17223577	17223626	1.00E-10	71.6
	Chr_07	84.31	21527132	21527174	8.00E-07	59
	Chr_09	91.38	8607622	8607565	1.00E-11	75.2
	Chr_09	94.29	54905757	54905791	1.00E-03	48.2
	Chr_05	89.66	6842151	6842094	1.00E-10	71.6
	Chr_03	85.96	3660977	3661025	4.00E-10	69.8
	Chr_02	89.83	5254404	5254346	4.00E-10	69.8
	Chr_08	86.54	23125711	23125664	6.00E-08	62.6
	Chr_01	86	28185062	28185013	8.00E-07	59
	Chr_01	83.64	8376194	8376242	9.00E-06	55.4
	Chr_01	85.11	28185083	28185041	3.00E-05	53.6
Sorghum bicolor	Chr07	78.85	770960	770629	6.00E-75	286
	Chr07	83.82	773166	773099	8.00E-10	69.8
	Chr07	83.33	771034	770983	2.00E-05	55.4
	Chr04	91.38	63425817	63425874	2.00E-11	75.2
	Chr04	83.33	66726864	66726825	6.00E-05	53.6
	Chr03	84.62	51323970	51323927	4.00E-07	60.8
	Chr03	82.14	677429	677484	5.00E-06	57.2

Species	Chromosome / Linkage group	Identity	Start	End	E value	Score
	Chr10	84	8588323	8588282	5.00E-06	57.2
	Chr01	84	18788701	18788742	5.00E-06	57.2
Zea mays	6	76.78	2147467	2147113	1.00E-64	253
	6	84.62	2147976	2147928	1.00E-05	57.2
	4	85.71	39738460	39738357	2.00E-22	113
	3	91.38	166465789	166465732	5.00E-11	75.2
	3	85.37	35357473	35357513	7.00E-03	48.2
	3	89.47	49150755	49150790	9.00E-02	44.6
	9	88.33	126257265	126257324	6.00E-10	71.6
	1	84.31	35047361	35047403	4.00E-06	59
	5	91.3	114809148	114809191	4.00E-06	59
	5	83.67	7631953	7631993	5.00E-05	55.4
	2	82	224596475	224596516	2.00E-04	53.6
	2	91.18	124715591	124715560	2.60E-02	46.4
	2	81.82	190474078	190474113	2.60E-02	46.4
	10	88.57	27620113	27620083	9.00E-02	44.6
Triticum aestivum	ta_iwgsc_5bs_v1_457869	91.07	6853	6906	5.00E-12	77
	ta_iwgsc_2dl_v1_9909120	90	2448	2391	5.00E-12	77
	ta_iwgsc_1al_v2_3975476	94.12	2246	2196	5.00E-12	77
	ta_iwgsc_5al_v1_2742183	87.1	1843	1786	2.00E-11	75.2
	ta_iwgsc_1bs_v1_3462460	91.38	2070	2013	2.00E-11	75.2
	ta_iwgsc_1as_v1_529860	91.38	2434	2377	2.00E-11	75.2
	ta_iwgsc_5dl_v1_4553403	89.29	15524	15575	6.00E-11	73.4
	ta_iwgsc_1dl_v1_2273792	85.25	690	742	6.00E-11	73.4
	ta_iwgsc_5bl_v1_10910316	85.96	8479	8535	2.00E-09	68
	ta_iwgsc_5bl_v1_10867123	85.71	3077	3030	2.00E-09	68
	ta_iwgsc_3b_v1_10759089	87.27	4333	4383	2.00E-09	68
	ta_iwgsc_1al_v2_3905383	86.89	3917	3857	2.00E-09	68
	ta_iwgsc_5dl_v1_4604052	91.11	10592	10636	3.00E-08	64.4
	ta_iwgsc_7bl_v1_6737043	86.79	1748	1800	1.00E-06	59
	ta_iwgsc_4as_v2_5930405	84.31	1747	1705	1.00E-06	59

Species	Chromosome / Linkage group	Identity	Start	End	E value	Score
	ta_iwgsc_4as_v2_5930405	83.67	1899	1859	2.00E-05	55.4
	ta_iwgsc_7dl_v1_2022790	84	1	42	4.00E-06	57.2
	ta_iwgsc_4dl_v3_14326198	83.67	14943	14903	2.00E-05	55.4
	ta_iwgsc_1as_v1_3296694	77.27	4063	4120	2.00E-05	55.4
	ta_iwgsc_4bs_v1_4898005	82	3775	3818	2.00E-04	51.8
	ta_iwgsc_4bl_v1_6903702	82.98	1	39	2.00E-04	51.8
	ta_iwgsc_4al_v2_7129828	81.63	13698	13738	2.00E-04	51.8
	ta_iwgsc_4al_v2_7068933	81.63	3780	3740	2.00E-04	51.8
	ta_iwgsc_6dl_v1_3290973	82.22	1621	1657	2.00E-03	48.2
	ta_iwgsc_3al_v1_4354342	82.22	303	339	2.00E-03	48.2
	ta_iwgsc_7dl_v1_3384295	80.43	3443	3482	2.70E-02	44.6
	ta_iwgsc_5bl_v1_10799316	84.21	268	305	9.60E-02	42.8
	ta_iwgsc_3b_v1_10758293	90.32	7732	7762	9.60E-02	42.8
	ta_iwgsc_1bl_v1_3906435	90.32	14966	14996	9.60E-02	42.8

Marker: UGEP68

Species	Chromosome/ Linkage group	Identity	Start	End	E value	Score
Brachypodium distachyon	Bd1	90.32	12435936	12435966	4.30E-02	42.8
Brachypodium stacei	Chr02	84.21	10417763	10417726	3.70E-02	42.8
	Chr01	90.32	14781653	14781623	3.70E-02	42.8
Oryza sativa	Chr6	90.24	9439234	9439194	3.00E-05	53.6
	Chr1	83.72	7653206	7653164	5.00E-03	46.4
	Chr2	84.78	33833253	33833208	1.70E-02	44.6
	Chr10	96.15	10183678	10183703	5.90E-02	42.8
Panicum hallii	Chr02	93.33	5088932	5088903	7.00E-03	46.4
Panicum virgatum	Chr08a	86.36	4086941	4086984	2.00E-03	50
Setaria italica	scaffold_9	73.56	20454095	20454176	4.00E-04	50
	scaffold_2	70.14	34374916	34374780	1.00E-03	48.2
	scaffold_2	70.97	26336417	26336325	6.40E-02	42.8

Species	Chromosome/ Linkage group	Identity	Start	End	E value	Score
	scaffold_7	91.18	35962831	35962798	1.80E-02	44.6
	scaffold_5	91.18	47252243	47252210	1.80E-02	44.6
Setaria viridis	Chr_08	91.18	1359907	1359874	1.80E-02	44.6
	Chr_09	72.84	20780885	20780960	6.20E-02	42.8
	Chr_09	88.57	37223282	37223248	6.20E-02	42.8
	Chr_02	70.97	25170067	25169975	6.20E-02	42.8
Sorghum bicolor	Chr07	81.63	1505713	1505761	3.00E-03	48.2
	Chr02	91.18	73026060	73026093	3.00E-03	48.2
	Chr02	83.33	68431916	68431957	3.30E-02	44.6
	super_12	80	2605268	2605219	3.30E-02	44.6
	Chr03	88.24	12247501	12247468	3.30E-02	44.6
	Chr01	80	36290476	36290427	3.30E-02	44.6
Zea mays	5	93.1	167822987	167822959	9.30E-02	44.6
	7	86.49	132631714	132631678	9.30E-02	44.6
	7	93.55	174888844	174888815	9.30E-02	44.6
	8	84.09	173259844	173259801	9.30E-02	44.6
Triticum aestivum	ta_iwgsc_6dl_v1_3240604	96.15	3031	3056	9.90E-02	42.8
	ta_iwgsc_6bs_v1_3017092	90.32	533	563	9.90E-02	42.8
	ta_iwgsc_5bl_v1_10926297	81.25	4742	4788	9.90E-02	42.8
	ta_iwgsc_5bl_v1_10867888	82.93	11252	11292	9.90E-02	42.8

Marker: UGEP90

Species	Chromosome/ Linkage group	Identity	Start	End	E value	Score
Brachypodium distachyon	Nil	-	-	-	-	-
Brachypodium stacei	Chr05	77.58	10784973	10784811	1.00E-26	123
	Chr01	84.09	21476513	21476556	8.00E-04	48.2
	Chr01	83.33	28079247	28079288	1.00E-02	44.6
	Chr03	93.33	8479568	8479539	3.00E-03	46.4
	Chr10	88.24	17533375	17533342	1.00E-02	44.6

**Species**	**Chromosome/ Linkage group**	**Identity**	**Start**	**End**	**E value**	**Score**
	Chr02	86.11	24045182	24045147	3.50E-02	42.8
Oryza sativa	Chr9	84.44	12267466	12267422	1.00E-03	48.2
	Chr10	80.85	2327300	2327254	1.60E-02	44.6
	Chr2	88.24	5427206	5427239	1.60E-02	44.6
Panicum hallii	Chr02	75.29	41957107	41956687	2.00E-61	241
	Chr02	85.48	41956623	41956563	3.00E-08	64.4
	Chr02	77.27	41956308	41956243	6.00E-04	50
	Chr05	88.64	20287358	20287317	6.00E-04	50
	Chr04	93.75	18659142	18659111	6.00E-04	50
	Chr09	88.37	8994083	8994123	2.00E-03	48.2
	Chr01	86.36	49027235	49027194	7.00E-03	46.4
	Chr08	87.8	19481897	19481859	2.40E-02	44.6
Panicum virgatum	contig69046	91.41	4464	4625	5.00E-55	221
	contig81907	88.71	128	188	1.00E-10	73.4
	contig81907	76.92	438	515	1.00E-04	53.6
	Chr02b	78.43	50644970	50644876	1.00E-10	73.4
	Chr02b	91.43	50644778	50644745	2.10E-02	46.4
	contig26001	85.45	3	54	1.00E-04	53.6
	contig182170	86.96	36	81	2.00E-03	50
	contig17785	84.44	9821	9781	2.00E-03	50
	Chr08b	84.44	18496290	18496246	2.00E-03	50
	Chr09a	84.09	22779192	22779153	6.00E-03	48.2
	Chr05a	88.37	19573516	19573556	6.00E-03	48.2
	Chr02a	83.33	26268678	26268632	6.00E-03	48.2
	contig68490	82	4158	4203	2.10E-02	46.4
	contig33627	86.36	7102	7061	2.10E-02	46.4
	contig329873	87.8	3	41	7.30E-02	44.6
	contig34098	87.8	7039	7001	7.30E-02	44.6

**Species**	**Chromosome/ Linkage group**	**Identity**	**Start**	**End**	**E value**	**Score**
Setaria italica	scaffold_2	75.55	28442614	28442280	6.00E-59	232
	scaffold_2	77.46	28441901	28441831	3.00E-05	53.6
	scaffold_2	79.69	28442188	28442125	1.00E-04	51.8
	scaffold_6	83.33	28961321	28961274	1.00E-04	51.8
	scaffold_6	83.33	29002641	29002594	1.00E-04	51.8
	scaffold_5	96.55	40548835	40548807	1.00E-03	48.2
Setaria viridis	Chr_02	75.55	27346682	27346348	6.00E-59	232
	Chr_02	77.46	27345969	27345899	3.00E-05	53.6
	Chr_02	79.69	27346256	27346193	1.00E-04	51.8
	Chr_06	83.33	28429784	28429737	1.00E-04	51.8
	Chr_05	96.55	39429734	39429706	1.00E-03	48.2
Sorghum bicolor	Chr02	87.34	56870685	56870529	1.00E-45	188
	Chr02	96.77	56869872	56869842	2.00E-04	51.8
	Chr02	76.67	56870247	56870188	9.00E-03	46.4
	Chr02	100	56870417	56870393	9.00E-03	46.4
	Chr03	88.37	5484283	5484241	6.00E-05	53.6
	Chr09	86.27	163556	163604	9.00E-03	46.4
	Chr09	87.8	57142030	57142068	3.20E-02	44.6
	Chr06	77.46	4691390	4691320	3.20E-02	44.6
Zea mays	2	84.85	177888273	177888436	6.00E-42	178
	2	73.39	177888568	177888672	7.00E-03	48.2
	7	81.33	100291343	100291507	5.00E-37	161
	7	76.83	100291681	100291762	4.00E-06	59
	7	90	100292189	100292228	6.00E-04	51.8
	6	78.64	123021408	123021307	1.00E-13	84.2
	10	85.71	141048469	141048428	2.00E-03	50
Triticum aestivum	ta_iwgsc_5bl_v1_10814677	74.58	2183	2357	2.00E-23	114
	ta_iwgsc_5bl_v1_10814677	90.62	3152	3183	2.70E-02	44.6

Species	Chromosome/ Linkage group	Identity	Start	End	E value	Score
	ta_iwgsc_5dl_v1_4529992	74.01	19671	19845	2.00E-22	111
	ta_iwgsc_5dl_v1_4529992	90.91	20519	20551	8.00E-03	46.4
	ta_iwgsc_3b_v1_10754810	88.24	11600	11567	2.70E-02	44.6
	ta_iwgsc_4dl_v3_14404266	78.95	17161	17107	9.50E-02	42.8
